# Effect of land-use changes on the abundance, distribution, and host-seeking behavior of *Aedes* arbovirus vectors in oil palm-dominated landscapes, southeastern Côte d’Ivoire

**DOI:** 10.1371/journal.pone.0189082

**Published:** 2017-12-07

**Authors:** Julien B. Z. Zahouli, Benjamin G. Koudou, Pie Müller, David Malone, Yao Tano, Jürg Utzinger

**Affiliations:** 1 Swiss Tropical and Public Health Institute, Basel, Switzerland; 2 University of Basel, Basel, Switzerland; 3 Centre Suisse de Recherches Scientifiques en Côte d’Ivoire, Abidjan, Côte d’Ivoire; 4 Unité de Formation et de Recherche Biosciences, Université Félix Houphouët-Boigny, Abidjan, Côte d’Ivoire; 5 Centre for Neglected Tropical Diseases, Liverpool School of Tropical Medicine, Liverpool, United Kingdom; 6 Unité de Formation et de Recherche Sciences de la Nature, Université Nangui-Abrogoua, Abidjan, Côte d’Ivoire; 7 Innovative Vector Control Consortium, Liverpool School of Tropical Medicine, Liverpool, United Kingdom; Institut Pasteur, FRANCE

## Abstract

**Background:**

Identifying priority areas for vector control is of considerable public health relevance. Arthropod-borne viruses (arboviruses) spread by *Aedes* mosquitoes are (re)emerging in many parts of the tropics, partially explained by changes in agricultural land-use. We explored the effects of land-use changes on the abundance, distribution, and host-seeking behavior of *Aedes* mosquitoes along a gradient of anthropogenic disturbance in oil palm-dominated landscapes in southeastern Côte d’Ivoire.

**Methodology:**

Between January and December 2014, eggs, larvae, pupae, and adults of *Aedes* mosquitoes were sampled in four types of macrohabitats (rainforest, polyculture, oil palm monoculture, and rural housing areas), using standard procedures (bamboo-ovitraps, metallic-ovitraps, larval surveys, and human-baited double-net traps). Immature stages were reared and adult mosquitoes identified at species level.

**Principal findings:**

A total of 28,276 *Aedes* specimens belonging to 11 species were collected. No *Aedes*-positive microhabitat and only four specimens of *Ae*. *aegypti* were found in oil palm monoculture. The highest abundance of *Aedes* mosquitoes (60.9%) was found in polyculture, while the highest species richness (11 species) was observed in rainforest. *Ae*. *aegypti* was the predominant *Aedes* species, and exhibited high anthropophilic behavior inflicting 93.0% of total biting to humans. The biting rate of *Aedes* mosquitoes was 34.6 and 7.2-fold higher in polyculture and rural housing areas, respectively, compared to rainforest. Three species (*Ae*. *aegypti*, *Ae*. *dendrophilus*, and *Ae*. *vittatus*) bit humans in polyculture and rural housing areas, with respective biting rates of 21.48 and 4.48 females/person/day. Unexpectedly, all three species were also feeding during darkness. *Aedes* females showed bimodal daily feeding cycles with peaks at around 08:00 a.m. and 05:00 p.m. Host-seeking activities were interrupted between 11:00 a.m. and 02:00 p.m. in rural housing areas, while no such interruption was observed in polyculture. Some rainforest-dwelling *Aedes* species displayed little preference to feed on humans.

**Conclusions:**

In southeastern Côte d’Ivoire, the agricultural land-use/land-cover changes due to the conversion of rainforest into oil palm monocultures influence the abundance, distribution, and host-seeking behaviors of anthropophagic and non-anthropophagic *Aedes* vectors. As a result, there is higher risk of humans to arbovirus transmission in polyculture and rural housing areas. There is a need for integrated vector management, including landscape epidemiology and ecotope-based vector control.

## Introduction

*Aedes* mosquito-transmitted arthropod-borne viruses (arboviruses) have (re)emerged from their sylvatic reservoirs of Africa and the Americas under landscape anthropization forces [1]. Indeed, arboviruses are dispersed globally, and they are responsible for various diseases [1]. Several *Aedes* species act as vectors of arboviral diseases, such as yellow fever, dengue, chikungunya, Rift valley fever, and Zika that are of considerable public health relevance [1]. The resurgence of these mosquito-borne diseases and their geographic expansion has long been associated with human-induced modifications of terrestrial ecosystems [2]. Identifying priority areas for integrated vector management (IVM) is crucial for public health because the ecology (e.g., abundance distribution, and behavior) of *Aedes* mosquito vectors is likely to alter with human-induced land-use changes, including deforestation, intensification of agriculture, and urbanization [2–4].

The expansion of tropical oil palm (*Elaesis guineensis*) plantations is a major driver of deforestation and threatens biodiversity, including arthropods [5, 6]. Wild palm trees have a life-span of up to 200 years, and an economic life-span of 25–30 years, after which trees are cut down and replaced with young palm plants. The planting density ranges from 120 to 160 palms/ha. Changes in land-use can result in the losses of *Aedes* mosquito habitats, hosts, and predators, which, in turn, affect the dynamics, abundance, oviposition, and host-seeking behaviors of vectors searching for alternative habitats and new blood-feeding sources [2]. In contrast, other cultivations such as rubber plantations, and plants with sheathing leaf axils (e.g., banana, bromeliads, and taro), and fruit husks (e.g., coconuts) can be important sources of *Aedes* mosquito breeding as they retain water for larval breeding [7, 8]. Additionally, containers used to supply water to animals and plants support *Aedes* mosquito larval growth [9]. Anthropogenic chemicals, such as pesticides (e.g., insecticides, fungicides, herbicides, and rodenticides), are drivers of changes in mosquito populations [10]. While the transformation of native rainforests into human settlements might destroy natural breeding sites of *Aedes*, it might result in an increase of artificial containers (e.g., tires, discarded containers, and water storage receptacles) that serve as microhabitats for immature *Aedes* [2]. Moreover, open areas directly exposed to sunlight that are created after the removal of natural vegetation accelerate mosquito development and survivorship [4, 8]. Tropical rainforests are rich in biodiversity, including *Aedes* that might breed in tree holes that are protected by foliage and contain microbial food sources for mosquito larvae [2, 7]. In addition, the diverse fauna in the rainforest [7] serves as blood sources for host-seeking *Aedes* females, thereby maintaining the circulation of arboviruses among non-human primates (sylvatic cycle) [11, 12]. Deforestation, forest-degradation, and forest-fragmentation have been associated with arbovirus emergence or re-emergence [11, 12]. The effects of these multiple anthropogenic changes in land-use on mosquito communities and the risk of disease transmission in the tropics may be further amplified by changing patterns of precipitation [2, 13].

In the southeastern part of Côte d’Ivoire, where large parts of rainforest have been converted into oil palm plantations, several outbreaks of yellow fever and dengue have been documented [14]. Yellow fever and dengue viruses have been associated with vectors such as *Ae*. *aegypti*, *Ae*. *africanus*, *Ae*. *furcifer*, *Ae*. *luteocephalus*, *Ae*. *opok*, and *Ae*. *vittatus* [15, 16]. At present, Côte d’Ivoire is the third largest African producer of palm oil with an annual production of about 1.8 million tons. Palm oil production generates 3.1% of the national gross domestic product (GDP) [17]. There are plans to enlarge the national production of palm oil, which might increase human-induced pressures on rainforest [18].

Meanwhile, there is a lack of knowledge on how agricultural land-use changes affect the ecology of *Aedes* vectors in oil palm-dominated landscapes of Côte d’Ivoire. It is important to deepen the understanding of this relationship to identify priority areas for IVM and to provide a better land-use strategy for the reduction of arboviral disease risks. Hence, our study aimed at assessing the effects of land-use changes on the ecology of *Aedes* mosquitoes among four major land-cover types (rainforest, polyculture, oil palm monoculture, and rural housing areas) derived from human-driven landscape transformation in large industrial oil palm areas in southeastern Côte d’Ivoire. We hypothesized that the abundance, distribution, oviposition, and host-seeking behaviors of *Aedes* mosquito species differ depending on the main landscape type.

## Methods

### Ethics statement

The study protocol was approved by the local health and administrative authorities of PALMCI, which manages the industrial oil palm plantations where our study was conducted. The management of PALMCI provided a field permit for mosquito sampling. Before starting the study, informed oral consent was provided by village leaders. In addition, all entomologic surveys and sample collections carried out on private lands or private residential areas were done with the permission and written informed consent of the residents.

The volunteers participating to the human-baited double-net trapping gave written, informed consent for their participation. They were between 21 and 45 years old, and were given a small remuneration for their participation. Volunteers were vaccinated against yellow fever and protected against malaria with medical prophylaxis. Participants were also offered the opportunity to receive free medical treatment when they showed any symptoms suspected to be caused by mosquito-borne diseases. Moreover, the volunteers were not directly exposed to mosquito females’ biting because they were protected by the inner nets of the double-net trap device. The volunteers who sampled *Aedes* mosquitoes for this study are among the authors, rather than being subjects of the study. This study did not involve endangered or protected species.

### Study area

**Fig 1:**
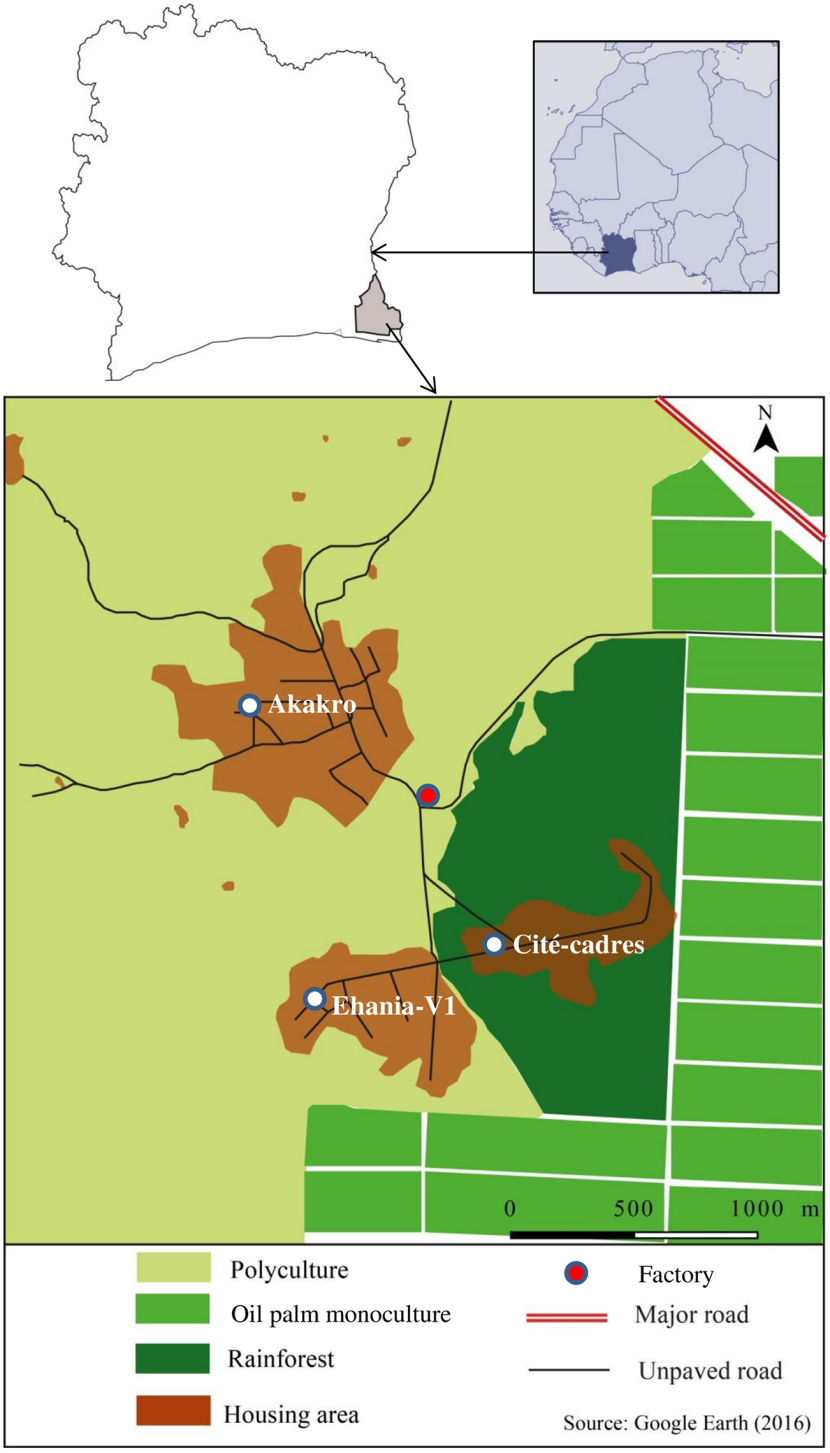
Location of the study areas in south-eastern Côte d’Ivoire. The study was carried out in the villages located in oil palm plantation areas belonging to the Sud-Comoé region. The study area covers the villages of Ehania-V1, Cité-cadre and Akakro situated at the interface between the industrial oil palm plantation and traditional agricultural smallholdings. The industrial exploitations are devoted to the monoculture of oil palm plantations (*Eleasis guineensis*) covering over 30,000 hectares managed by an integrated agro-industrial unit of PALMCI. In the industrial part, a primary rainforest of over 100 ha has been preserved intact and forbidden of any human activities. In the traditional lands, the agricultural exploitation systems are polycultures comprising oil palm trees, rubber trees, banana, taro, bromeliads, and cocoa growing in the same space. Several small villages averaging 20 people are dispersed in these smallholdings.

The study was carried out in the Sud-Comoé region (geographic coordinates 5° 28’ N latitude, 3° 12’ W longitude) located in the southeastern part of Côte d’Ivoire (Fig 1). The estimated human population in the Sud-Comoé region is 642,000 with people mainly living in rural settings. The economic activities are primarily based on subsistence agriculture. Additionally, there is some industrial exploitations of oil palm monocultures (approximately 30,000 ha), managed by PALMCI. Chemical products (i.e., insecticides, fungicides, and herbicides) are intensively used for oil palm plantation and crop protection [19]. The natural vegetation mostly constitutes of rainforest. Several small villages are dispersed across the landscape. The rainforest and traditional agriculture host trees, bamboo, and diverse animal species (primates, and birds).

The climate in the study area is characterized by high temperature and precipitation with two rainy seasons. The seasons are mainly distinguished by rainfall. The main rainy season extends from May to July, while the shorter rainy season lasts from October to November, with distinct dry seasons in between. The average annual precipitation ranges from 1,200 to 2,400 mm. The annual average temperature and relative humidity are around 26.5°C and 80–90%, respectively.

Our study was conducted in the Aboisso department, covering some 625 km^2^ and an estimated population of 21,300 people, many of whom are employees of PALMCI. The workers leave the villages in the morning to work in the plantations and return in the afternoon.

### Study design

The study area was divided into 10 blocks around eight villages of Ehania (Ehania-V1-8), Cité-Cadre, and Akakro. In each block, four types of macrohabitats of roughly equal size were classified as rainforest, polyculture, oil palm monoculture, and rural housing areas based on the land-cover features defined by remote sensing and geospatial analyses (Table 1 and Figures A-D in S1 Fig). The blocks with the villages of Ehania-V1, Cité-Cadre, and Akakro were selected for this study (Fig 1).

**Table 1:** Classification of *Aedes* mosquito habitats sampled in oil palm-dominated landscapes in southeastern Côte d’Ivoire from January to October 2014.

	Term	Definition
**I**	**Macrohabitat**^1^	**Landscape covering specific floristic area and presenting ecological or phyto-geographic aspects that are roughly homogeneous**
A	Rainforest^a^	Area covered with dense forest showing natural ecosystems with strong canopy coverage and comprising big trees, creepers, fixed masses of bamboo (*Bambusae*), and wild vertebrate animals such as primates, birds, and reptiles
B	Polyculture^a^	Area covered with a mosaic of oil palm trees (*Eleasis guineensis*) mixed with other multiple crops composed of the plants of several industrial crops, such as rubber (*Hevea brasiliensis*), cocoa (*Theobroma cacao*), coffee (*Coffea* spp.), papaya (*Carica papaya*), coconuts (*Cocos* spp.), and avocado (*Persea Americana*), and food-crops such as bananas (*Musa* spp.), taro (*Colocasia* spp.), bromeliads (*Ananas comosus*), yam (*Dioscorea* spp.), maize (*Zea mays*), and cassava (*Manihot esculenta*) growing in the same space. Natural trees, fixed masses of bamboo (*Bambusae*), and degraded or secondary forest relicts are dispersed in several places in the area
	Oil palm monoculture^a^	Area covered uniquely with the monoculture of oil palm trees (*Eleasis guineensis*)
D	Rural housing areas^a^	Area covered with human-inhabited space comprising buildings such as houses, markets, hospitals, schools, and other social structures
**II**	**Microhabitat**^1^	**Containers that might hold water and serve as breeding sites for *Aedes* mosquito larvae**
**II.1**	**Naturally-occurring microhabitat**^2^	**Containers created without or by indirect intervention of humans**
E	Natural tree hole^b^	Rot and pan holes of different shapes and volume located up to 2 m above the ground level
F	Bamboo hole^b^	Cut of fixed masses of bamboo (*Bambusae*)
G	Natural plant leaf^b^	Sheathing leaf axils from plants such as *Sanseviera* spp. and *Xanthosoma* spp., and sheets from *Thaumatococcus daniellii* fallen on the floor
H	Other natural microhabitat^b^	Non-ligneous containers such as snail shells and rock holes
**II.2**	**Agriculturally-occurring microhabitat**^2^	**Containers created by growing crops cultivated by humans**
I	Crop fruit husk^b^	Skins of coconuts (*Cocos* spp.) and cocoa (*Theobroma cacao*)
J	Crop flower^b^	Flowers of bananas (*Musa* spp.)
K	Crop leaf^b^	Sheathing leaf axils from plants such as bromeliads (*Ananas comosus*), taros (*Colocasia* spp.), and bananas (*Musa* spp.), and fallen sheets on the floor
L	Cultivated plant hole	Growing plant holes of different shapes and volume located up to 2 m above the ground level such as papaya (*Carica papaya*), coffee (*Coffea* spp.), avocado (*Persea Americana*), and cocoa (*Theobroma cacao*)
**II.3**	**Man-made microhabitat**^2^	**Containers created by direct intervention of humans**
M	Crop collection container^b^	Containers such as ceramic, cemented, glass, plastic, and metallic receptacles used to collect crops such as rubber latex collection cups
N	Husbandry watering container^b^	Containers such as ceramic, cemented, glass, plastic, and metallic receptacles used to store water for watering plant or animal husbandry
O	Discarded container^b^	Discarded cans, tires, tarps, broken bottles, buckets, shoes, calabashes, mortars, building tools, and debris of abandoned cars and machines
P	Household water container^b^	Containers such as ceramic, cemented, glass, plastic, and metallic receptacles used to store potable water or collect rainwater for drinking, cooking, or washing

^1^: habitat class,

^a^: macrohabitat type,

^2^: microhabitat category,

^b^:microhabitat sub-category.

Eggs, larvae, pupae, and adults of *Aedes* mosquitoes were sampled every month during 12 cross-sectional surveys from January to December 2014. There were four defined macrohabitats and we used metallic-ovitraps, bamboo-ovitraps, larvae surveys, and human-baited double-net traps for mosquito collection (Figures A-D in S2 Fig).

### *Aedes* mosquito egg collection

*Aedes* mosquito eggs were collected monthly using 30 bamboo-ovitraps and 30 metallic-ovitraps during the 12 cross-sectional surveys in each macrohabitat. Bamboo-ovitraps were made of cut bamboo, while metallic-ovitraps were made of a tin can cut to imitate natural and artificial breeding sites of *Aedes* mosquitoes, respectively. Metallic-ovitraps were painted black, while bamboo-ovitraps were left unpainted. Both ovitrap types had a volume of 400 cm^3^ and were filled to ¾ with water. The water was a mix of distilled water, rainwater, and a 10% hey infusion with *Panicum maximum* to increase the attractiveness of the ovitraps [20]. A 5 cm x 7 cm x 0.3 cm paddle made of hardboard served with its rough surface as an oviposition substrate and was plunged into each container and left for one week during each of the 12 surveys.

### Microhabitat surveys and *Aedes* spp. larval sampling

In a preliminary survey, existing larval breeding sites, such as natural and artificial cavities or containers with a potential to contain water, were defined as microhabitats for *Aedes* larvae. Based on this preliminary survey, microhabitats were classified into three categories and 12 sub-categories depending on their occurring process and use (Table 1 and Figures E-P in S1 Fig). We sampled up to 30 microhabitats of each of the 12 sub-category types among each macrohabitat.

Microhabitats were examined monthly, over a 12-month period (January-December 2014), for the presence of water and immature stages of mosquitoes. Whenever mosquito larvae and/or pupae were present, the content of microhabitat was completely removed using the following equipment: flexible rubber tube connected to a manual suction pump, ladles, and pipettes. Immature forms of *Aedes* and other non-*Aedes* mosquitoes such as *Anopheles* spp., *Culex* spp., *Eretmapodites* spp., and *Toxorhynchites* spp. were sampled and recorded separately. The *Aedes* mosquito immatures were counted and classified as young larvae (1–2 instar), old larvae (3–4 instar), and pupae. The predacious larvae of mosquitoes, such as *Cx*. *tigripes*, *Eretmapodites* spp., and *Toxorhynchites* spp., were removed from the samples and preserved separately to avoid predation on the other species. The microhabitats sampled were refilled to their initial volume with the original water, and topped up with distilled water or rainwater according to their flooding mechanism. The presence of shade, predators, and plant leaves in the microhabitats were recorded.

### *Aedes* adult abundance and host-seeking behavior surveillance

Adult mosquitoes were sampled using four human-baited double-net traps in each macrohabitat type for three consecutive days from 04:00 a.m. to 08:00 p.m. during 12 monthly cross-sectional surveys. A double-net trap was a combination of two untreated nets: an inner, smaller net that protected the human bait and an outer, larger net with two openings on each of the four sides which allowed the entry of mosquitoes yet precluded their exit [21, 22]. For each double-net trap, there was a pair of persons: one person was located inside the small net and served as bait to attract mosquitoes. The other person was located outside the double-net device and collected the mosquitoes trapped within the outer net, once every hour. Each trap was monitored by two teams of two persons each that took turns beginning at 12:00 a.m.

### Laboratory treatment procedures

All mosquito samples were stored separately in plastic boxes and transferred in a cool-box to a nearby field laboratory. In the laboratory, mosquito larvae were reared until they became adult. In order to minimize mortality, a maximum of 20 larvae were placed in 200 ml plastic cups, filled with 150 ml distilled water and covered with netting. Larvae of *Aedes* and other mosquitoes were fed each morning between 07:00 and 08:00 a.m. with Tetramin baby fish food. Predacious larvae (e.g., *Toxorhynchites* spp. and *Cx*. *tigripes*) were fed with larvae from additionally sampled mosquitoes from the study area. Emerging adults and collected adult mosquitoes were identified to species level using readily available morphological keys [20, 23]. As larval mortalities were low, the proportion of mosquito species was estimated on the basis of emerging adults. Adult specimens were stored by species and recorded in an entomology collection database.

### Statistical analysis

The *Aedes*-positive index (PI) was calculated as the percentage of bamboo-ovitraps, metallic-ovitraps, microhabitats, or human-baited double-net traps which collected or held at least one egg, larva, pupa, or adult *Aedes* mosquito (numerator) among the total bamboo-ovitraps, metallic-ovitraps, wet microhabitats, or double-net traps found (denominator), respectively. The *Aedes* microhabitat positive proportion (PPM) refers to the percentage of each *Aedes*-positive microhabitat type (numerator) among the total *Aedes*-positive containers (denominator) in a specific macrohabitat. The *Aedes* microhabitat positive proportion (PPSA) was calculated as the percentage of each *Aedes*-positive microhabitat type (numerator) among the total *Aedes*-positive containers (denominator) in the study area. The proportions of *Aedes* species were calculated as percentage of specimens among *Aedes* fauna. We used Fisher’s exact test to determine the relationship between species composition and the macro- and microhabitats. Fisher’s exact test was employed because expected numbers of specimens were equal or less than five. *Aedes* species richness was expressed as the number of collected species in each study area [24, 25] and compared using a one-way analysis of variance (ANOVA), followed by Bonferroni’s correction. The species diversity, dominance, and community similarity of *Aedes* mosquitoes in the study area and among the macrohabitats were estimated by Shannon index (H) (1), Simpson index (D) (2), and Sorenson’s coefficient (CC) (3) [24, 25], and analyzed by Kruskal-Wallis test because the log-transformed data exhibited significant deviations from normality. The abundance of *Aedes* mosquitoes was the number of specimens per species and calculated as the mean numbers of specimens per bamboo-ovitrap, metallic-ovitrap, wet microhabitat and human-baited double-net trap according to sampling methods. The *Aedes* females’ biting rate was expressed as the mean number of female specimens per person per day. The number of persons was equal to the number of participants used as attractants during human-baited double-net trap sampling. The bamboo-ovitrap, metallic-ovitrap, and human-baited double-net trap data were tested using repeated measures approaches in generalized linear mixed models (GLMM), in order to take into account possible interactions between the variables “macrohabitats” and “month” [26]. We used repeated measures approaches in GLMM framework because the bamboo-ovitrap, metallic-ovitrap, and human-baited double-net trap were repeatedly installed in the same sampling location over time (months). The microhabitat survey data were analyzed using a generalized linear model (GLM) approach. To account for overdispersion due to excessive number of zeroes, the data were log-transformed [log (number of specimens + 1)]. A significance level of 5% was set for statistical testing. All statistical analyses were conducted using Stata version 14.0 (Stata Corporation; College Station, TX, United States of America).

The formulas of the biodiversity indicators were [24, 25]:

$$Shannon\;index(H)=-\sum_{i=1}^{s}p_{i}\;lnp_{i}$$ (1)

$$Simpson\;index(D)=1/\sum_{i=1}^{s}{p_{i}}^{2}$$ (2)

$$Sorenson\prime s\;coefficient\;(CC)=\frac{2C}{S1+S2}$$ (3)

where *p*_*i*_ is the proportion (n/N) of specimens of one particular species *i* found (n) divided by the total number of specimens found (N), ln is the natural log, ∑ is the sum of the calculations, *s* is the number of species, *C* is the number of species that the two communities have in common, *S1* is the total number of species found in community 1, and *S2* is the total number of species found in community 2. The Shannon index (H) is an information statistic index which assumes that all species are represented in a sample and are randomly sampled. Note that, the higher the value of H, the higher the species diversity; while the lower the value of H, the lower the species diversity. The Simpson index (D) is a dominance index as it gives more weight to common or dominant species and assumes that a few rare species with only a few representatives will not affect the diversity. The higher the value of D, the higher the species abundance; whereas the lower the value of H, the lower the species abundance. Sorenson’s coefficient (CC) gives information on community similarity and helps to know how much two communities have overlap or similarity. CC ranges from 0 to 1. The closer the value is to 1, the more the communities have species in common; complete community overlap is equal to 1; and complete community dissimilarity is equal to 0.

## Results

### Mosquito species composition

Table 2 shows the species composition of adult mosquitoes collected as eggs, larvae, pupae, and adults using bamboo-ovitrap, metallic-ovitrap, larval survey, and human-baited double-net trap methods. A total of 30,449 mosquito specimens were collected, comprising different medically important genera, such as *Aedes*, *Anopheles*, *Culex*, *Mansonia*, and predatory larvae of *Eretmapodites* and *Toxorhynchites*. For any sampling method, *Aedes* mosquitoes dominated the fauna, representing 92.9% of the total fauna with 11 species. The proportions, sex, and the numbers of mosquito species varied substantially between sampling methods.

**Table 2:** Species composition of mosquitoes sampled in oil palm-dominated landscapes in southeastern Côte d’Ivoire from January to December 2014.

Genus	Species	Bamboo-ovitrap				Metallic-ovitrap				Larval survey				Double-net trap				Total			
		F	M	T	%	F	M	T	%	F	M	T	%	F	M	T	%	F	M	T	%
*Aedes*	*Ae*. *aegypti*	1,382	1,343	2,725	8.9	2,052	1,952	4,004	13.1	3,909	3,742	7,651	25.1	6,735	1,286	8,021	26.3	14,078	8,323	22,401	73.6
	*Ae*. *africanus*	163	167	330	1.1	199	193	392	1.3	120	141	261	0.9	59	9	68	0.2	541	510	1,051	3.5
	*Ae*. *dendrophilus*	410	408	818	2.7	528	481	1,009	3.3	405	384	789	2.6	302	58	360	1.2	1,645	1,331	2,976	9.8
	*Ae*. *fraseri*	16	11	27	0.1	27	38	65	0.2	16	21	37	0.1	0	0	0	0.0	59	70	129	0.4
	*Ae*. *furcifer*	41	35	76	0.2	62	70	132	0.4	145	122	267	0.9	23	3	26	0.1	271	230	501	1.6
	*Ae*. *lilii*	26	16	42	0.1	13	15	28	0.1	9	5	14	0.0	0	0	0	0.0	48	36	84	0.3
	*Ae*. *luteocephalus*	42	50	92	0.3	67	49	116	0.4	27	27	54	0.2	0	0	0	0.0	136	126	262	0.9
	*Ae*. *metallicus*	13	16	29	0.1	44	49	93	0.3	25	23	48	0.2	0	0	0	0.0	82	88	170	0.6
	*Ae*. *opok*	13	30	43	0.1	9	1	10	0.0	8	7	15	0.0	0	0	0	0.0	30	38	68	0.2
	*Ae*. *palpalis*	6	6	12	0.0	19	13	32	0.1	55	62	117	0.4	3	1	4	0.0	83	82	165	0.5
	*Ae*. *vittatus*	29	13	42	0.1	98	80	178	0.6	57	38	95	0.3	119	35	154	0.5	303	166	469	1.5
	**Total**	**2,141**	**2,095**	**4,236**	**13.9**	**3,118**	**2,941**	**6,059**	**19.9**	**4,776**	**4,572**	**9,348**	**30.7**	**7,241**	**1,392**	**8,633**	**28.4**	**17,276**	**11,000**	**28,276**	**92.9**
*Anopheles*	*An*. *pharoensis*	0	0	0	0.0	0	0	0	0.0	8	2	10	0.0	0	0	0	0.0	8	2	10	0.0
	*An*. *gambiae*	0	0	0	0.0	0	0	0	0.0	39	48	87	0.3	19	2	21	0.1	58	50	108	0.4
	*An*. *ziemani*	0	0	0	0.0	0	0	0	0.0	0	0	0	0.0	1	0	1	0.0	1	0	1	0.0
	**Total**	**0**	**0**	**0**	**0.0**	**0**	**0**	**0**	**0.0**	**47**	**50**	**97**	**0.3**	**20**	**2**	**22**	**0.1**	**67**	**52**	**119**	**0.4**
*Culex*	*Cx*. *nebulosus*	19	27	46	0.2	52	43	95	0.3	15	19	34	0.1	6	0	6	0.0	92	89	181	0.6
	*Cx*. *poicilipes*	32	36	68	0.2	29	41	70	0.2	73	54	127	0.4	48	5	53	0.2	182	136	318	1.0
	*Cx*. *quinquefasciatus*	74	62	136	0.4	89	71	160	0.5	218	176	394	1.3	56	11	67	0.2	437	320	757	2.5
	*Cx*. *tigripes*	3	4	7	0.0	13	6	19	0.1	79	95	174	0.6	3	0	3	0.0	98	105	203	0.7
	**Total**	**128**	**129**	**257**	**0.8**	**183**	**161**	**344**	**1.1**	**385**	**344**	**729**	**2.4**	**113**	**16**	**129**	**0.4**	**809**	**650**	**1,459**	**4.8**
*Eretmapodites*	*Er*. *chrysogaster*	87	69	156	0.5	76		76	0.2	112	97	209	0.7	48	14	62	0.2	323	180	503	1.7
	**Total**	**87**	**69**	**156**	**0.5**	**76**	**0**	**76**	**0.2**	**112**	**97**	**209**	**0.7**	**48**	**14**	**62**	**0.2**	**323**	**180**	**503**	**1.7**
*Mansonia*	*Ma*. *africana*	0	0	0	0.0	0	0	0	0.0	0	0	0	0.0	6	0	6	0.0	6	0	6	0.0
	*Ma*. *uniformis*	0	0	0	0.0	0	0	0	0.0	0	0	0	0.0	2	1	3	0.0	2	1	3	0.0
	**Total**	**0**	**0**	**0**	**0.0**	**0**	**0**	**0**	**0.0**	**0**	**0**	**0**	**0.0**	**8**	**1**	**9**	**0.0**	**8**	**1**	**9**	**0.0**
*Toxorhynchites*	*Tx*. *brevipalpis*	0	0	0	0.0	0	0	0	0.0	47	36	83	0.3	0	0	0	0.0	47	36	83	0.3
	**Total**	**0**	**0**	**0**	**0.0**	**0**	**0**	**0**	**0.0**	**47**	**36**	**83**	**0.3**	**0**	**0**	**0**	**0.0**	**47**	**36**	**83**	**0.3**
**Total**	**Abundance**	**2,356**	**2,293**	**4,649**	**15.3**	**3,377**	**3,102**	**6,479**	**21.3**	**5,367**	**5,099**	**10,466**	**34.4**	**7,430**	**1,425**	**8,855**	**29.1**	**18,530**	**11,919**	**30,449**	**100**
	**No. of species**	**16**				**16**				**19**				**15**				**22**			

F: female, M: male, T: total, %: percentage.

### Distribution of *Aedes* immature stages across macrohabitats

**Fig 2:**
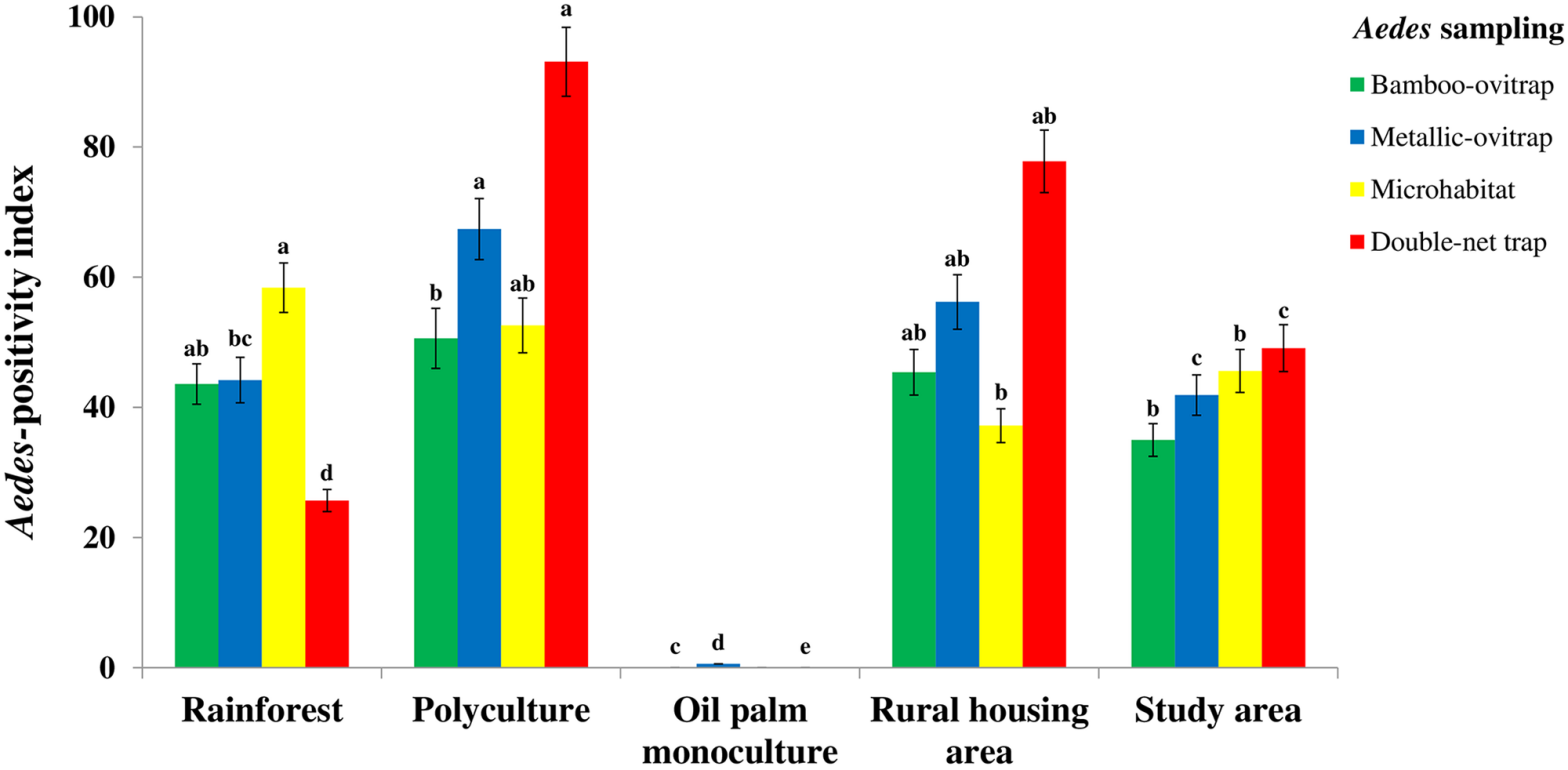
*Aedes* mosquito species occurrence among the macrohabitats in oil in oil palm-dominated landscapes in southeastern Côte d’Ivoire from January to December 2014. Error bars represent the standard error (SE). Letters indicate the results of the GLMM. Groups that do not share the same letter for the same sampling method are significantly different.

Fig 2 and Table 3 illustrate immature *Aedes* species occurrence, stratified by macrohabitats. Overall, the study area showed variable *Aedes*-positivity indices, with PI values of 35.0% (482/1,378) in the bamboo-ovitraps, 41.9% (577/1,377) in metallic-ovitraps, and 45.6% (801/1,756) in the microhabitats. The highest *Aed*es-positivity indices in the bamboo-ovitraps (177/350; PI = 50.6%) and in the metallic-ovitraps (232/344; PI = 67.4%) were found in the polyculture environment. Conversely, GLMM indicated that *Aedes*-positivity indices were significantly lower in oil palm monoculture compared to the other macrohabitats (p <0.05) (S1 Table).

**Table 3:** *Aedes* mosquito positivity patterns among the macrohabitats, and the study area in southeastern Côte d’Ivoire from January to December 2014.

Term	Macrohabitat												Study area		
	Rainforest			Polyculture			Oil palm monoculture			Rural housing area					
	n_1_	n_2_	PI	n_1_	n_2_	PI	n_1_	n_2_	PI	n_1_	n_2_	PI	n_1_	n_2_	PI
Bamboo-ovitrap^1^	346	151	43.6	350	177	50.6	343	0	0.0	339	154	45.4	1,378	482	35.0
Metallic-ovitrap^2^	344	152	44.2	344	232	67.4	349	2	0.6	340	191	56.2	1,377	577	41.9
Microhabitat^3^	161	94	58.4	737	388	52.6	0	0	NA	858	319	37.2	1,756	801	45.6
Naturally-occurring microhabitat^3^	161	94	58.4	148	94	63.5	0	0	NA	47	10	21.3	356	198	55.6
Natural tree hole^3^	54	45	83.3	42	33	78.6	0	0	NA	4	1	25.0	100	79	79.0
Bamboo hole^3^	51	38	74.5	29	21	72.4	0	0	NA	13	4	30.8	93	63	67.7
Natural plant leaf^3^	52	9	17.3	29	7	24.1	0	0	NA	11	0	0.0	92	16	17.4
Other natural microhabitat^3^	4	2	50.0	48	33	68.8	0	0	NA	19	5	26.3	71	40	56.3
Agriculturally-occurring microhabitat^3^	0	0	NA	314	96	30.6	0	0	NA	49	6	12.2	363	102	28.1
Crop fruit husk^3^	0	0	NA	91	47	51.6	0	0	NA	26	6	23.1	117	53	45.3
Crop flower^3^	0	0	NA	68	3	4.4	0	0	NA	16	0	0.0	84	3	3.6
Crop leaf^3^	0	0	NA	96	11	11.5	0	0	NA	0	0	NA	96	11	11.5
Cultivated plant hole^3^	0	0	NA	59	35	59.3	0	0	NA	7	0	0.0	66	35	53.0
Man-made microhabitat^3^	0	0	NA	275	198	72.0	0	0	NA	762	303	39.8	1,037	501	48.3
Crop collection container^3^	0	0	NA	57	33	57.9	0	0	NA	6	2	33.3	63	35	55.6
Husbandry watering container^3^	0	0	NA	51	30	58.8	0	0	NA	229	159	69.4	280	189	67.5
Discarded container^3^	0	0	NA	167	135	80.8	0	0	NA	167	105	62.9	334	240	71.9
Household water container^3^	0	0	NA	0	0	NA	0	0	NA	360	37	10.3	360	37	10.3
Double-net trap^4^	144	37	25.7	144	134	93.1	144	0	0.0	144	112	77.8	576	283	49.1

n_1_: numbers of bamboo-ovitraps recovered^1^, metallic-ovitraps recovered^2^, wet microhabitats^3^, or double-net traps installed^4^, n_2_: numbers of *Aedes*-positive bamboo-ovitraps^1^, numbers of *Aedes*-positive metallic-ovitraps^2^, *Aedes*-positive microhabitats^3^, or *Aedes*-positive double-net traps^4^, PI: *Aedes*-positivity index. PI is expressed as percentage (%).

Microhabitat *Aedes*-positivity indices widely varied from one macrohabitat to another (Table 3 and S3 Fig). No *Aedes*-positive microhabitats were found in oil palm monoculture. In contrast, the highest *Aedes*-microhabitat positivity index was estimated for the rainforest (94/161; PI = 58.4%), followed by the polyculture (388/737; PI = 52.6%), and the rural housing areas (319/858; PI = 37.2%). In the rural housing areas, husbandry watering containers were often infested with *Aedes* larvae (159/229; PI = 69.4%), and reached a PI of 86.4% (19/22) in December 2014 during the long dry season. In the polyculture site, the highest *Aedes*-positivity index (135/167; PI = 80.8%) was observed among the discarded containers.

Table 4 shows the proportions of each type of *Aedes*-positive microhabitats among the whole *Aedes*-positive microhabitats in each macrohabitat. In the rainforest, all the *Aedes*-positive breeding sites (94/94; PPM = 100%) were naturally occurring microhabitats, while 95.0% (303/319, PPM = 95.0%) of *Aedes*-positive microhabitats were man-made containers in the rural housing areas. The polyculture macrohabitat had substantial proportions of all *Aedes*-positive microhabitat types, with PPM of 24.2% (94/388) of naturally-occurring, 24.8% (96/388) of agriculturally-occurring, and 51.0% (198/388) of man-made microhabitats. In the study area, *Aedes*-positive breeding sites were dominated by man-made microhabitats (501/801; PPSA = 62.6%), followed by naturally-occurring microhabitats (198/801; PPSA = 24.7%), and agricultural microhabitats (102/801; PPSA = 12.7%) (Table 4 and S4 Fig). Overall, apart from the oil palm monocultures, *Aedes* microhabitat positivity indices were higher during the dry season (January, February, November, and December), in the other macrohabitats and the study area (S5 Fig). Conversely, the highest proportions of *Aedes*-positive microhabitats were recorded during the rainy seasons (June, July, and October; see S6 Fig).

**Table 4:** Proportion (%) of each *Aedes*-positive microhabitat type among *Aedes*-positive microhabitats, macrohabitats, and study area in southeastern Côte d’Ivoire from January to December 2014.

Term	Macrohabitat												Study area	
	Rainforest			Polyculture			Oil palm monoculture			Rural-housing area				
	n	PPM	PPSA	n	PPM	PPSA	n	PPM	PPSA	n	PPM	PPSA	n	PPSA
Naturally-occurring microhabitat	94	100.0	11.7	94	24.2	11.7	0	NA	0.0	10	3.1	1.2	198	24.7
Natural tree hole	45	47.9	5.6	33	8.5	4.1	0	NA	0.0	1	0.3	0.1	79	9.9
Bamboo hole	38	40.4	4.7	21	5.4	2.6	0	NA	0.0	4	1.3	0.5	63	7.9
Natural plant leaf	9	9.6	1.1	7	1.8	0.9	0	NA	0.0	0	0.0	0.0	16	2.0
Other natural microhabitats	2	2.1	0.2	33	8.5	4.1	0	NA	0.0	5	1.6	0.6	40	5.0
Agriculturally-occurring microhabitat	0	0.0	0.0	96	24.8	12.0	0	NA	0.0	6	1.9	0.7	102	12.7
Crop fruit husk	0	0.0	0.0	47	12.1	5.9	0	NA	0.0	6	1.9	0.7	53	6.6
Crop flower	0	0.0	0.0	3	0.8	0.4	0	NA	0.0	0	0.0	0.0	3	0.4
Crop leaf	0	0.0	0.0	11	2.8	1.4	0	NA	0.0	0	0.0	0.0	11	1.4
Cultivated plant hole	0	0.0	0.0	35	9.0	4.4	0	NA	0.0	0	0.0	0.0	35	4.4
Man-made microhabitat	0	0.0	0.0	198	51.0	24.7	0	NA	0.0	303	95.0	37.8	501	62.6
Crop collection container	0	0.0	0.0	33	8.5	4.1	0	NA	0.0	2	0.6	0.2	35	4.4
Husbandry watering container	0	0.0	0.0	30	7.7	3.7	0	NA	0.0	159	49.8	19.9	189	23.6
Discarded container	0	0.0	0.0	135	34.8	16.9	0	NA	0.0	105	32.9	13.1	240	30.0
Household water container	0	0.0	0.0	0	0.0	0.0	0	NA	0.0	37	11.6	4.6	37	4.6
**Total**	**94**	**100**	**11.7**	**388**	**100**	**48.5**	**0**	**NA**	**0.0**	**319**	**100**	**39.8**	**801**	**100**

n: number of *Aedes*-positive microhabitats, PPM: proportions of *Aedes*-positive microhabitat type among the whole *Aedes*-positive microhabitats in each macrohabitat, PPSA: proportions of *Aedes*-positive microhabitat type among the whole *Aedes*-positive microhabitats in the study area. PPM and PPSA are expressed as percentage (%).

The frequency of microhabitats with shade, plant leaves, and predators varied among the macrohabitats. The highest proportions of shaded microhabitats (n = 607; 96.9%), and microhabitats with plant leaves (92.6%) were found in the rainforest. Wet microhabitats containing at least one of the predatory larvae of *Toxorhynchites* spp., *Eretmapodites* spp., and *Cx*. *tigripes* mosquitoes were also mostly encountered in the rainforest (n = 161; 63.4%). The polyculture area also hosted higher numbers of microhabitats with shade (n = 2,117; 54.5%), plant leaves (n = 2,117; 59.6%), and predators (n = 737; 29.9%), compared to the rural housing areas.

### *Aedes* species distribution, biodiversity, and dynamics

Table 5 presents the geographic distribution and biodiversity of *Aedes* species among the macrohabitats in the study area. *Ae*. *aegypti* was the predominant species in the study area (n = 28,276; 79.2%). *Ae*. *aegypti* was also the most abundant species among *Aedes* mosquitoes collected in the polyculture, rural housing areas, and rainforest macrohabitats, with 49.2% (n = 28,276), 25.7%, and 4.3% of total fauna, respectively. Other *Aedes* species such as *Ae*. *dendrophilus* (10.5%), *Ae*. *africanus* (3.7%), *Ae*. *furcifer* (1.8%), and *Ae*. *vittatus* (1.7%), represented more than 1% of the total *Aedes* fauna in the study area. However, *Ae*. *africanus* (3.4%) showed its highest abundance in the rainforest, whereas the highest proportion of *Ae*. *dendrophilus* (7.6%) and *Ae*. *furcifer* (1.2%) were found in the polyculture area. The proportion of *Ae*. *dendrophilus* was above 1% in the rural housing area.

**Table 5:** *Aedes* species distribution and biodiversity among macrohabitats in oil palm-dominated landscapes in southeastern Côte d’Ivoire between January and December 2014.

Species	Macrohabitat								Study area	
	Rainforest		Polyculture		Oil palm monoculture		Rural housing area			
	Number	%	Number	%	Number	%	Number	%	Number	%
*Ae*. *aegypti*	1,213	4.3	13,903	49.2	4	0.01	7,281	25.7	22,401	79.2
*Ae*. *africanus*	948	3.4	61	0.2	0	0.0	42	0.1	1,051	3.7
*Ae*. *dendrophilus*	544	1.9	2,150	7.6	0	0.0	282	1	2,976	10.5
*Ae*. *fraseri*	129	0.5	0	0.0	0	0.0	0	0.0	129	0.5
*Ae*. *furcifer*	24	0.1	352	1.2	0	0.0	125	0.4	501	1.8
*Ae*. *lilii*	53	0.2	31	0.1	0	0.0	0	0.0	84	0.3
*Ae*. *luteocephalus*	96	0.3	158	0.6	0	0.0	8	0.0	262	0.9
*Ae*. *metallicus*	25	0.1	126	0.4	0	0.0	19	0.1	170	0.6
*Ae*. *opok*	24	0.1	34	0.1	0	0.0	10	0.0	68	0.2
*Ae*. *palpalis*	35	0.1	130	0.5	0	0.0	0	0.0	165	0.6
*Ae*. *vittatus*	24	0.1	289	1	0	0.0	156	0.6	469	1.7
Abundance (no. of specimens)	3,115	11.0	17,234	60.9	4	0.01	7,923	28.0	28,276	100
Species richness (no. of species)	11		10		1		8		11	
Species diversity (Shannon index H)	1.54		0.74		0.00		0.40		0.84	
Species dominance (Simpson index D)	0.28		0.67		1.00		0.85		0.64	
Community similarity (Sorenson’s coefficient CC)	1.00		0.95		0.17		0.84		1.00	
	0.95		1.00		0.18		0.89		0.95	
	0.17		0.18		1.00		0.22		0.17	
	0.84		0.89		0.22		1.00		0.84	
	1.00		0.95		0.17		0.84		1.00	

%: proportion of *Aedes* specimens calculated as percentages (%). In each row, a macrohabitat with a Sorenson’s coefficient CC of 1 was used as a reference to calculate the Sorenson’s coefficients for the other macrohabitats.

*Aedes* species number, diversity (F = 17.12; df = 3, p <0.05), and dominance (F = 11.04; df = 3, p <0.05) varied among the study area and the macrohabitats (Table 5). The highest *Aedes* species richness (n = 11) and the highest species diversity (Shannon index H = 1.54) were observed in the rainforest, while oil palm monoculture exhibited the poorest diversity with one species and null Shannon index. The rural housing areas displayed significantly higher *Aedes* species dominance (Simpson index D = 0.085) compared with the rainforest (Simpson index D = 0.28), the study area (Simpson index D = 0.64), and the polyculture (Simpson index D = 0.67). The community similarity of *Aedes* species between the macrohabitats also significantly altered (χ^2^ = 13.36; df = 3, p <0.05) (Table 5). According to Sorenson’s coefficient (CC = 1), *Aedes* mosquito community in the study area were similar to those inhabiting the rainforest. Compared with the rainforest, the polyculture showed the highest community similarity with Sorenson’s coefficient of 0.95, followed by the rural-housing areas with a Sorenson’s coefficient of 0.85. In contrast, the *Aedes* communities in the rainforest and oil palm monoculture showed with 0.17 the lowest value for the Sorenson’s coefficient.

Table 6 indicates *Aedes* species abundance among the macrohabitats in the study area. No *Aedes* eggs, larvae, pupae, or adults were collected in the oil palm monoculture using bamboo-ovitrap, larval survey, and double-net trap methods, except four eggs sampled with the metallic-ovitraps. However, higher mean numbers (mean ± standard error) of *Aedes* specimens with 2.32 ± 0.07 eggs/bamboo-ovitrap/week, 4.18 ± 0.07 eggs/metallic-ovitrap/week, and 26.01 ± 0.12 adults/double-net trap/day were found in the polyculture. The mean number in bamboo-ovitrap deployed in oil palm monoculture was significantly lower than the rainforest (Z = 1.96, p <0.05) and rural housing areas (Z = 2.06, p <0.05) (S2 Table). The mean numbers of *Aedes* eggs collected using metallic-ovitrap were significant different between the oil palm monoculture and the rainforest (Z = -2.04, p = 0.041) (S3 Table), and between the polyculture and the rainforest (Z = -3.45, p = 0.001) (S4 Table).

**Table 6:** *Aedes* mosquito abundance patterns in macrohabitats, and the study area in southeastern Côte d’Ivoire between January and December 2014.

Term	Macrohabitat												Study area		
	Rainforest			Polyculture			Oil palm monoculture			Rural housing area					
	n_1_	n_2_	Mean ± SE	n_1_	n_2_	Mean ± SE	n_1_	n_2_	Mean ± SE	n_1_	n_2_	Mean ± SE	n_1_	n_2_	Mean ± SE
Bamboo-ovitrap^1^	346	1,018	1.28 ± 0.06	350	1,899	2.32 ± 0.07	343	0	0	339	1,319	1.73 ± 0.06	1,378	4,236	1.13 ± 0.03
Metallic-ovitrap^2^	344	1,198	1.44 ± 0.06	344	2,830	4.18 ± 0.07	349	4	0.01 ± 0.004	340	2,027	2.72 ± 0.07	1,377	6,059	1.61 ± 0.03
Microhabitat^3^	607	671	0.36 ± 0.03	2,117	5,339	0.60 ± 0.02	0	0	NA	1,497	3,338	0.63 ± 0.03	4,221	9,348	0.57 ± 0.02
Naturally-occurring microhabitat^3^	607	671	0.36 ± 0.03	435	1,537	0.80 ± 0.06	0	0	NA	191	53	0.09 ± 0.03	1,233	2,261	0.45 ± 0.03
Natural tree hole^3^	92	372	1.87 ± 0.12	82	688	2.40 ± 0.18	0	0	NA	46	8	0.05 ± 0.05	220	1,068	1.48 ± 0.09
Bamboo hole^3^	189	257	0.48 ± 0.06	89	377	0.95 ± 0.14	0	0	NA	56	18	0.11 ± 0.06	334	652	0.52 ± 0.05
Natural plant leaf^3^	283	33	0.05 ± 0.02	111	54	0.14 ± 0.05	0	0	NA	28	0	0	422	87	0.07 ± 0.02
Other natural microhabitat^3^	43	9	0.08 ± 0.06	153	418	0.69 ± 0.09	0	0	NA	61	27	0.15 ± 0.07	257	454	0.43 ± 0.06
Agriculturally-occurring microhabitat^3^	0	0	NA	1,118	1,001	0.22 ± 0.02	0	0	NA	275	51	0.05 ± 0.02	1,393	1,052	0.19 ± 0.02
Crop fruit husk^3^	0	0	NA	338	556	0.41 ± 0.05	0	0	NA	98	51	0.14 ± 0.06	436	607	0.35 ± 0.04
Crop flower^3^	0	0	NA	266	16	0.02 ± 0.01	0	0	NA	54	0	0	320	16	0.02 ± 0.01
Crop leaf^3^	0	0	NA	360	75	0.06 ± 0.02	0	0	NA	89	0	0	449	75	0.05 ± 0.01
Cultivated plant hole^3^	0	0	NA	154	354	0.69 ± 0.08	0	0	NA	34	0	0	188	354	0.54 ± 0.07
Man-made microhabitat^3^	0	0	NA	564	2,801	1.50 ± 0.06	0	0	NA	1,031	3,234	0.98 ± 0.03	1,595	6,035	1.15 ± 0.03
Crop collection container^3^	0	0	NA	141	454	0.83 ± 0.10	0	0	NA	39	5	0.07 ± 0.05	180	459	0.63 ± 0.08
Husbandry watering container^3^	0	0	NA	63	303	1.99 ± 0.16	0	0	NA	272	1,362	2.47 ± 0.07	335	1,665	2.37 ± 0.06
Discarded container^3^	0	0	NA	360	2,044	1.74 ± 0.07	0	0	NA	360	1,560	1.20 ± 0.07	720	3,604	1.46 ± 0.05
Household water container^3^	0	0	NA	0		NA	0	0	NA	360	307	0.24 ± 0.04	360	307	0.24 ± 0.04
Double-net trap^4^	144	228	0.71 ± 0.7	144	7,166	26.01 ± 0.12	144	0	0	144	1,239	4.89 ± 0.10	576	8,633	3.06 ± 0.07

n_1_: number of recovered bamboo-ovitraps^1^, or number of recovered metallic-ovitraps^2^, or microhabiats^3^, or double-net trap^4^; n_2_: number of eggs, larvae, or adults of *Aedes* collected; SE: standard error of the mean numbers. Mean was mean number of *Aedes* eggs per bamboo-ovitrap^1^, mean number of *Aedes* eggs per metallic-ovitrap^2^, mean number of *Aedes* larvae per microhabitat^3^; or mean number of *Aedes* adults per double-net trap^4^. The units are egg/bamboo-ovitrap/week for bamboo-ovitraps^1^, egg/metallic-ovitrap/week for metallic-ovitraps^2^, larvae/microhabitat for microhabitats^3^, and adult/trap/day for double-net traps^4^.

GLMM revealed that the mean numbers of *Aedes* eggs were significantly lower in oil palm monoculture than the other macrohabitats (p <0.05) (S5 Table). The rural housing areas (0.63 ± 0.03 larvae/microhabitat) and the polyculture (0.60 ± 0.02 larvae/microhabitat) showed higher means of *Aedes* larvae compared with the other macrohabitats. In the rainforest, the tree holes were the most *Aedes*-inhabited habitats, with 1.87 ± 0.12 larvae/microhabitat. The rainforest was free of any agricultural and man-made microhabitats, while the polyculture macrohabitat hosted all types of microhabitats, except for household water containers. In the rural housing areas, the water containers were the most important producers of *Aedes* larvae with a mean of 2.47 ± 0.07 larvae/microhabitat. In the study area, the discarded containers also exhibited high ability to harbor *Aedes* immatures, with a mean number of 1.46 ± 0.05 larvae/microhabitat (Table 6).

**Fig 3:**
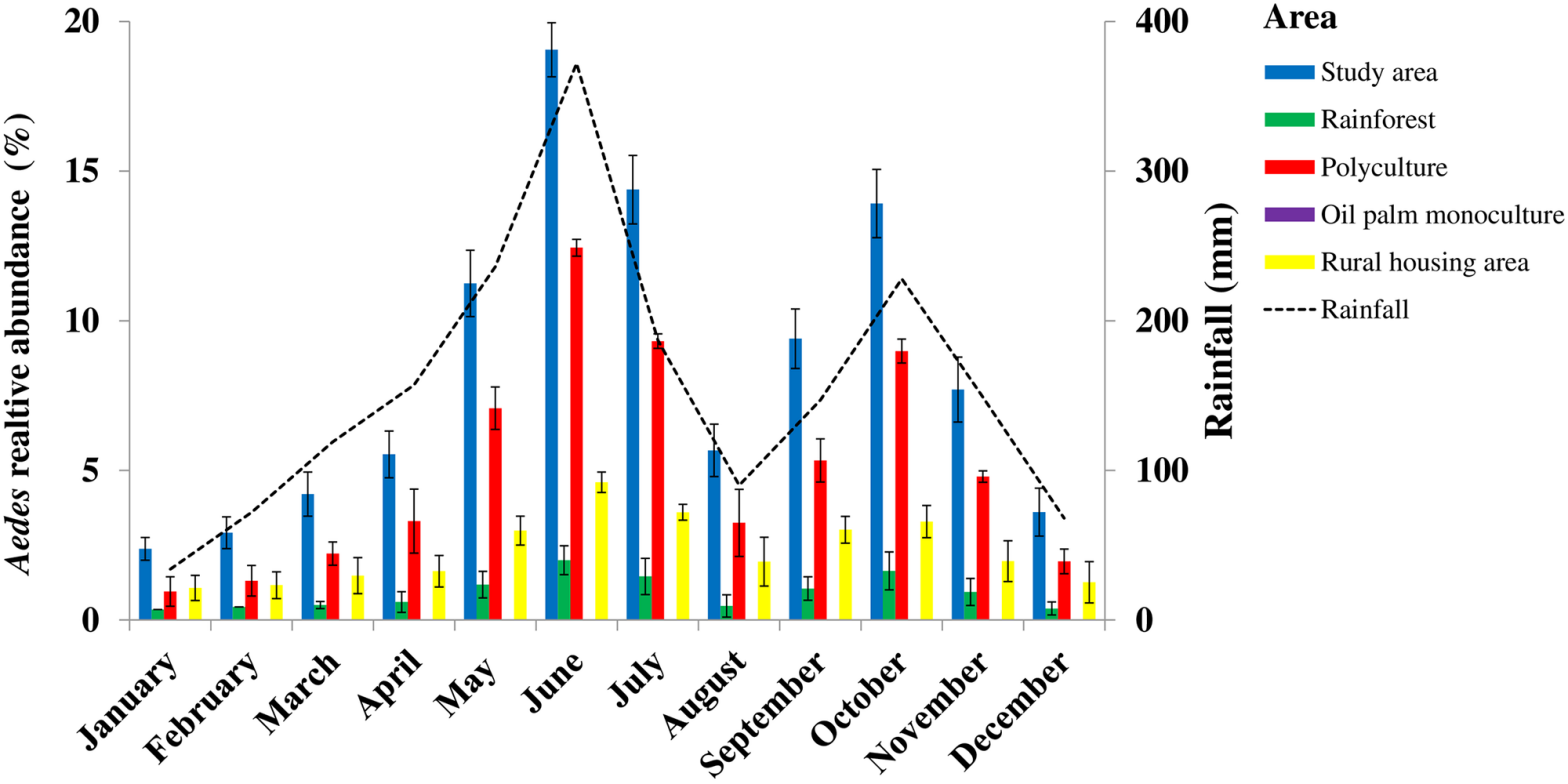
Monthly variations in the abundance of *Aedes* mosquitoes in oil palm-dominated landscapes in southeastern Côte d’Ivoire from January to December 2014. Error bars represent the standard error (SE).

Fig 3 shows the seasonal dynamics of whole *Aedes* species populations, sampled as eggs, larvae, pupae, and adults, over time among the macrohabitats in the study area. In the study area and macrohabitats, *Aedes* species abundance varied as a function of precipitation over time. *Aedes* abundance reached the first series of peaks in June, during the long rainy season, proportions of 19.1% (n = 28,276) in the study area, 12.4% in the polyculture, 4.6% in the rural housing areas, 2.0% in the rainforest, and 0.01% in oil palm monoculture. The second series of peaks occurred in October, during the short rainy season, with 13.9% in the study area, 9.0% in the polyculture, 3.3% in the rural housing areas, and 1.6% in the rainforest.

### Adult *Aedes* females’ host-seeking behaviors

The mean biting rate of *Aedes* females was estimated at 2.76 ± 0.07 females/person/day in the study area (S6 Table). The highest mean biting rates were found in the polyculture macrohabitat (21.48 ± 0.12 females/person/day), followed by the rural housing areas (4.48 ± 0.10 females/person/day), and the rainforest (0.62 ± 0.60 females/person/day). Hence, the polyculture, the rural housing areas, and the whole study area increased *Aedes* vector biting rate by factors of 34.6 (21.48/0.62), 7.2 (4.48/0.62), and 4.5 (2.76/0.62) compared with the rainforest, respectively. However, no biting *Aedes* females were collected in the oil palm monoculture. GLMM revealed significant differences in the mean biting rates comparing rainforest with polyculture (Z = 2.47, p = 0.014), and rainforest with housing areas (Z = 2.37, p = 0.018) (S3 Table). Over 93.0% (n = 7,241) of biting was inflicted by *Ae*. *aegypti*. Conversely, no females of several other species such as *Ae*. *fraseri*, *Ae*. *lilii*, *Ae*. *luteocephalus*, *Ae*. *metallicus*, and *Ae*. *opok* were found in the human-baited double-net device (Table 2).

**Fig 4:**
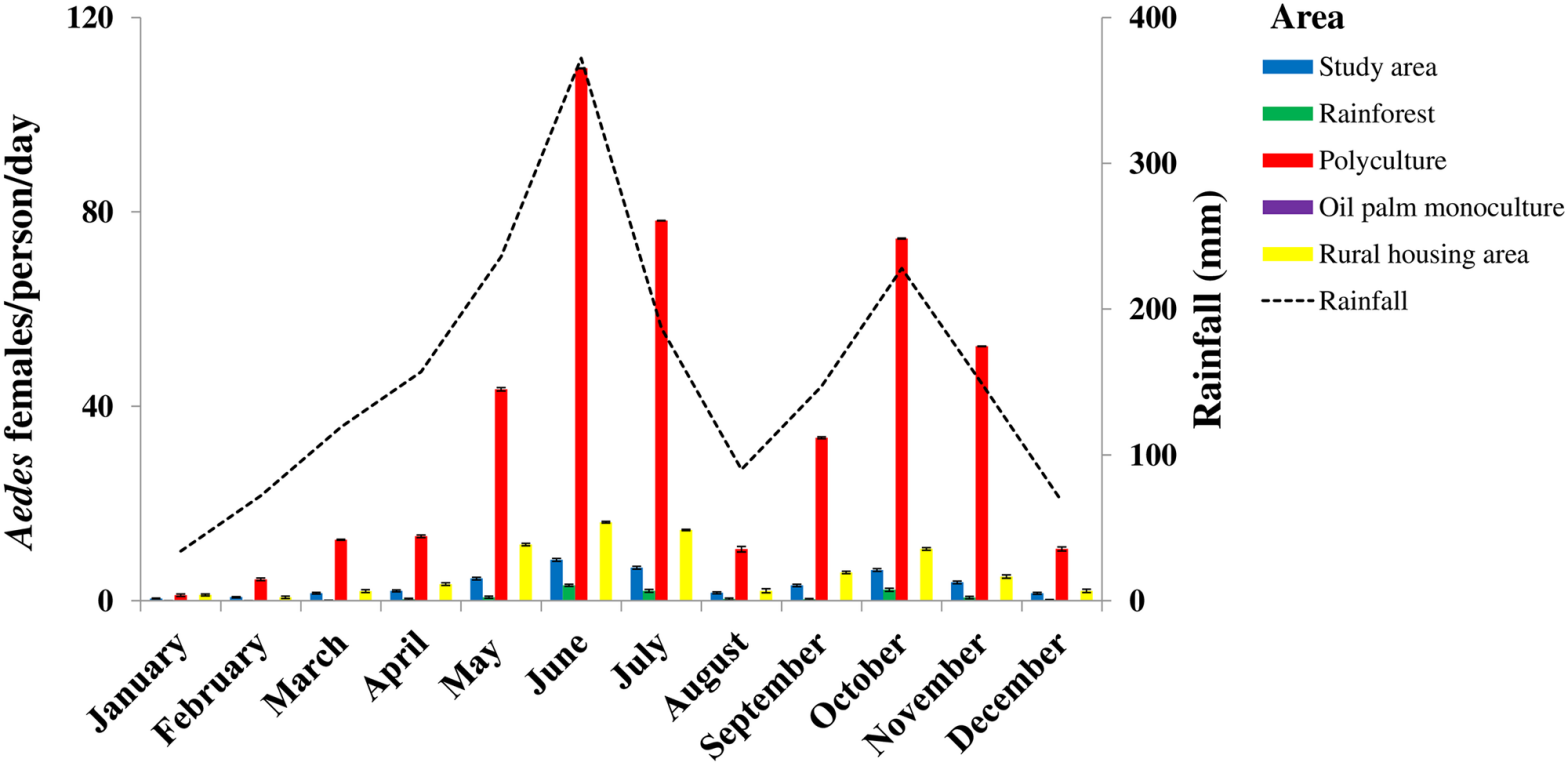
Monthly variations in *Aedes* mosquito females’ host-seeking activities in oil palm-dominated landscapes in southeastern Côte d’Ivoire from January to December 2014. Error bars represent the standard error (SE).

Fig 4 presents the seasonal dynamics of *Aedes* host-seeking in the study area and the macrohabitats. GLMM indicated that the biting rates of *Aedes* females significantly varied over time (p <0.05) (S5 Table) with a peak observed in June during the long rainy season and in October during the short rainy season across all macrohabitats, except for the oil palm monoculture (Fig 3). The major biting rate peaks of *Aedes* females averaged 109.54 ± 0.07 females/person/day in the polyculture, 16.14 ± 0.17 females/person/day in the rural housing area, 8.44 ± 0.30 females/person/day in the study area, and 3.18 ± 0.24 females/person/day in the rainforest in June. The second most important biting rates occurred in October with 74.5 ± 0.10 females/person/day in the polyculture, 10.7 ± 0.27 females/person/day in the rural-housing areas, 6.33 ± 0.29 females/person/day in the study area, and 2.27 ± 0.32 females/person/day in the rainforest.

**Fig 5:**
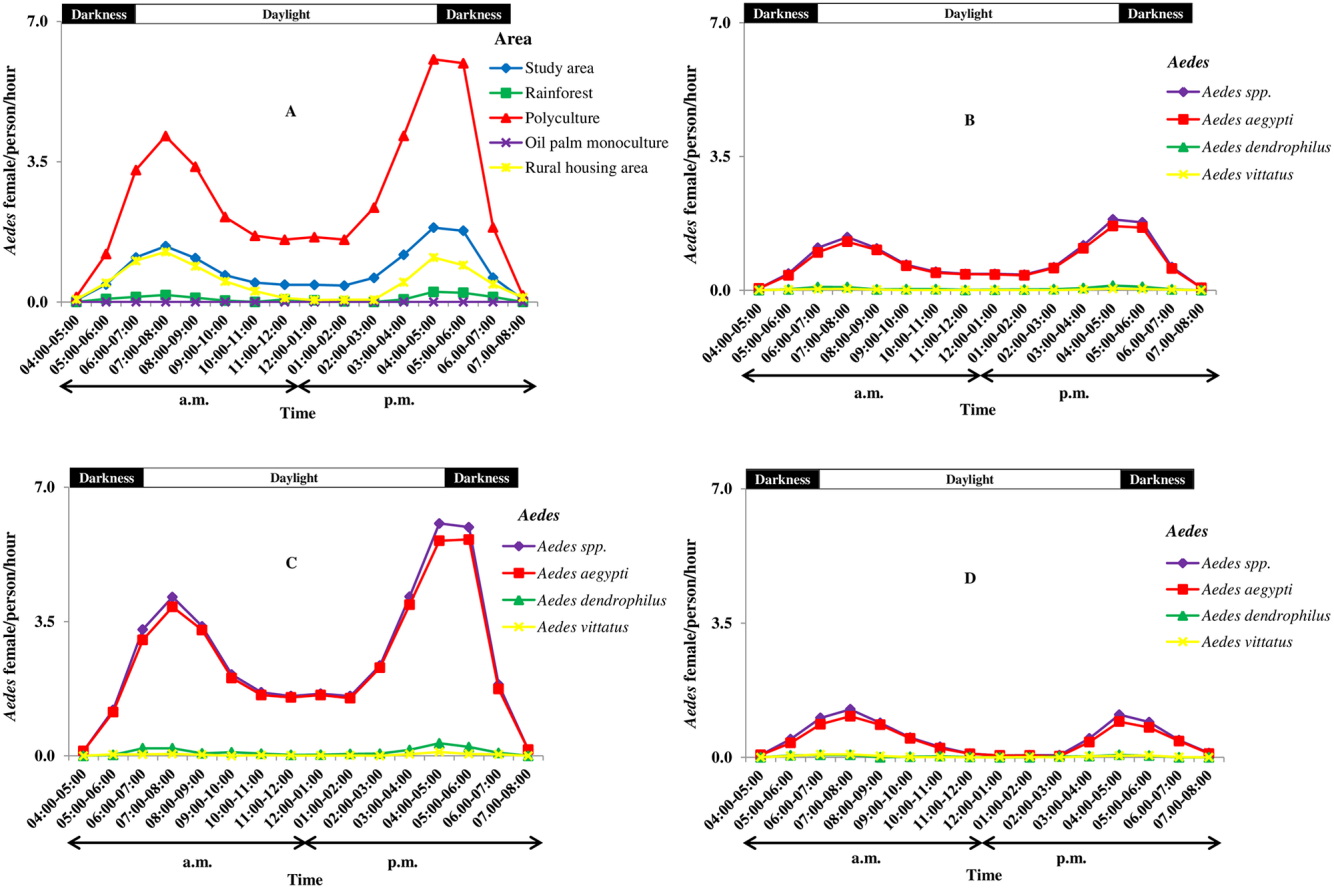
Nycthemeral dynamics of *Aedes* mosquito females’ host-seeking activities in oil palm-dominated landscapes in southeastern Côte d’Ivoire from January to December 2014. A: All species in all the macrohabitats and the study area, B: Prevalent *Aedes* species (> 1%) in the study area, C: Prevalent *Aedes* species (> 1%) in the polyculture, D: Prevalent *Aedes* species (> 1%) in the rural-housing areas.

Fig 5 shows the daily host-seeking activity cycles of *Aedes* mosquito females in the study area and across the different macrohabitats. *Aedes* females fed from 04:00 a.m. to 08:00 p.m., covering daytime (06:00 a.m. to 6:00 p.m.), and darkness (04:00 a.m. to 06:00 a.m. and 06:00 p.m. to 08:00 p.m.) in all macrohabitats, except in the oil palm monoculture (Fig 5A). The biting cycles showed two peaks, with the main peak observed between 04:00 p.m. and 05:00 p.m. and a lower peak between 07:00 a.m. and 08:00 a.m. *Ae*. *aegypti*, *Ae*. *dendrophilus*, and *Ae*. *vittatus* followed the same host-seeking patterns (Fig 5A) with stronger human biting intensity in *Ae*. *aegypti* in the study area (Fig 5B), the polyculture (Fig 5C), and the rural housing areas (Fig 5D). In contrast to these similarities, there was also some dissimilarity in that host-biting activity was interrupted from 11:00 a.m. to 02:00 p.m. in the rural housing areas but continued in polyculture macrohabitat (Fig 5A).

## Discussion

**Fig 6:**
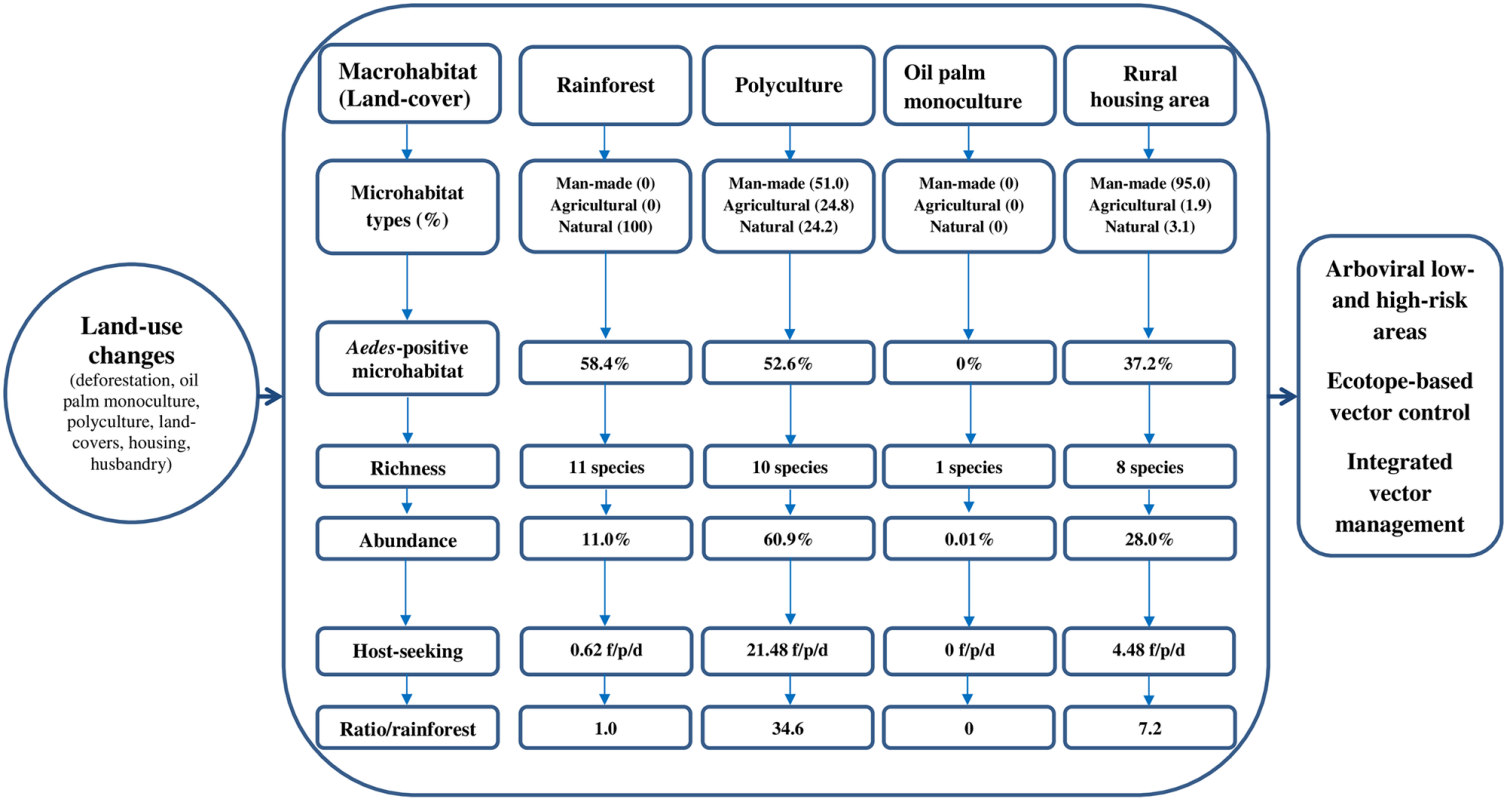
Synthesis of how agricultural land-use changes affect the dynamics of *Aedes* mosquitoes in oil palm-planted areas in southeastern Côte d’Ivoire. f/p/d: female/person/day. Overall, there was a lack of *Aedes* microhabitats and species in the oil palm monoculture. In contrast, the highest abundance of *Aedes* mosquitoes was found in the polyculture. The rural housing area also hosted substantial numbers of *Aedes* mosquitoes. Conversely, the highest *Aedes* species richness was observed in the rainforest where the preference of *Aedes* females to feed on humans was very little. As a result, the polyculture and the rural areas increased *Aedes* vectors’ biting rates by 34.6 and 7.2 times compared with the original rainforest, respectively.

**Fig 7:**
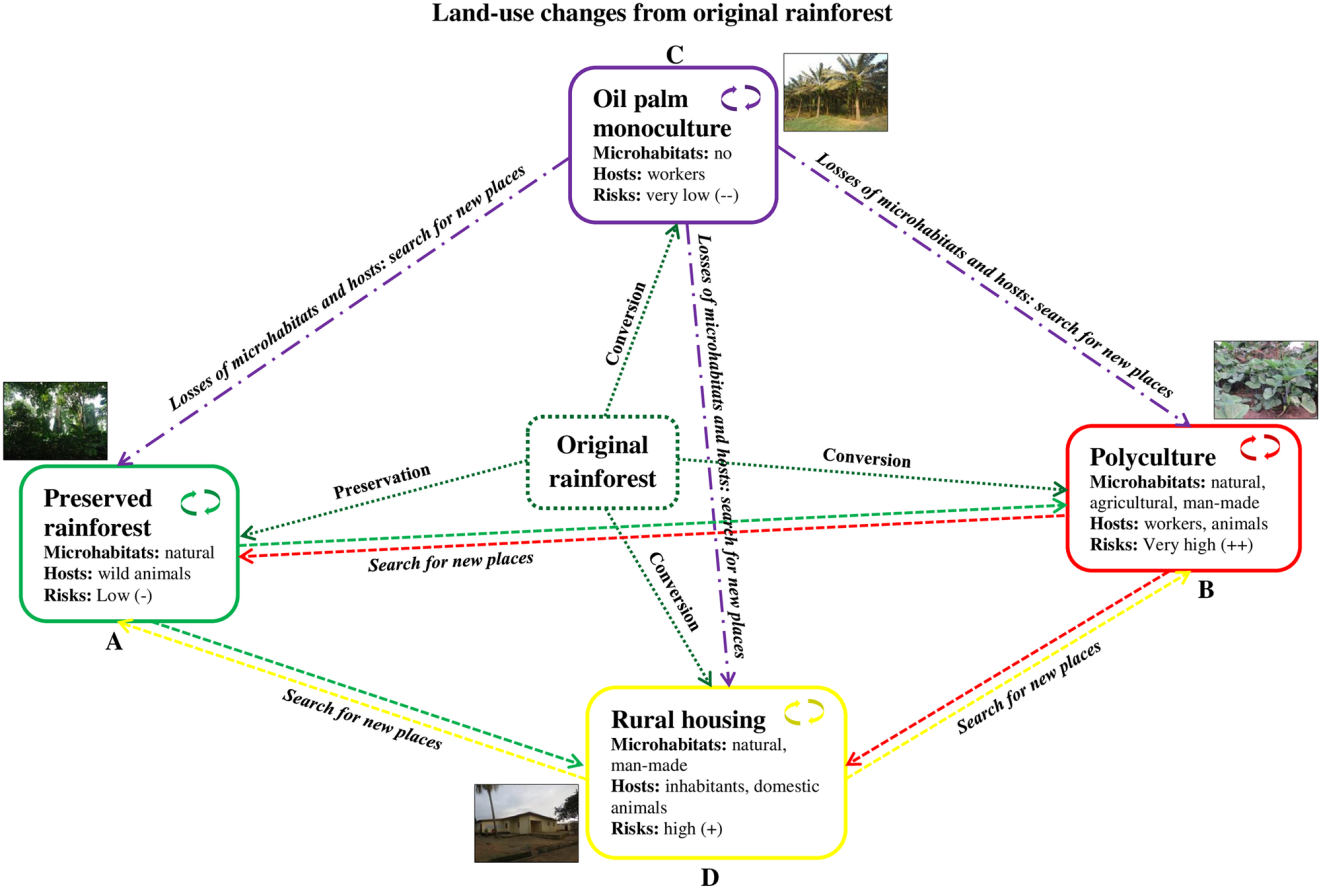
Effects of land-use changes on distribution of *Aedes* mosquitoes and arboviruses’ transmission risks in oil palm-dominated landscapes in southeastern Côte d’Ivoire. Human-induced land-use changes into the original tropical rainforests for their conversion in large industrial oil palm plantations have resulted in changes in land-covers creating four ecologically distinct macrohabitats: preserved rainforest (A), polyculture (B), oil palm monoculture (C), and rural housing area (D). The conversion of the original rainforests into large oil palm monoculture has led to the losses of the microhabitats and hosts of forest-dwelling *Aedes* mosquitoes thus increasing ecological pressure for searching alternative microhabitats and hosts in the three other macrohabitats, preserved rainforest, polyculture, and rural housing areas. *Aedes* mosquitoes found new microhabitats as anthropogenic containers abundantly encountered in the rural housing area and polyculture where humans (inhabitants and workers) were usually present thus resulting in higher abundance of vectors and high-risks of arboviruses’ transmission in these areas. In contrast, the arboviral transmission risks were very low in the oil palm monoculture due to the lack *Aedes* mosquitoes, and low in the rainforest due to the low anthropophagy of forest-dwelling *Aedes* species.

Our study revealed no *Aedes*-positive microhabitats and only four specimens of *Ae*. *aegypti* in oil palm monocultures, coupled with high *Aedes* species richness in the rainforest, and high biting rates in polyculture and rural housing areas over a 12-month period in southeastern Côte d’Ivoire. As identifying priority areas for IVM is of considerable importance for public health [3, 27], this study examined–for the first time–the effects of land-use changes on *Aedes* mosquito abundance, distribution, and human host seeking behavior in oil palm-dominated landscapes of yellow fever and dengue foci in the southeastern part of Côte d’Ivoire. Our data showed that *Aedes* mosquito species displayed several significant differences in community composition, distribution, and host-seeking behavior across different land-covers, with the highest species richness observed in rainforest, highest species numbers in the polyculture macrohabitats, the lowest species richness and numbers in oil palm monoculture, and stronger anthropophagic behaviors in the polyculture and rural housing areas (Fig 6 and S6 Table). Such distributional differences in *Aedes* vectors are likely to shape the distributions of arboviral disease transmission risks between landscapes, with low-risk and high-risk areas (Fig 7).

The following points are offered for discussion. First, holistically, our study yielded high species richness and high numbers of mosquitoes, with the dominance of medically important *Aedes* species in areas that have undergone anthropogenic land-use changes due to oil palm plantations. Several *Aedes* species (e.g., *Ae*. *aegypti*, *Ae*. *africanus*, *Ae*. *furcifer*, *Ae*. *luteocephalus*, *Ae*. *opok*, and *Ae*. *vittatus*) are known vectors for viral infections, including yellow fever, dengue, chikungunya, and Zika in Côte d’Ivoire [15, 16] and Senegal [7, 28, 29]. The high *Aedes* species diversity is consistent with previous studies conducted in distinct landscapes in rural areas of Senegal [7, 28, 29]. This could be due to the heterogeneity of landscapes (rainforest, polyculture, oil palm monoculture, and housing areas) that possibly provide a wide range of larval habitats, resting and mating places, and nectar and blood-food sources [7, 28].

Second, we used diverse sampling methods (i.e., bamboo-ovitraps, metallic-ovitraps, larval surveys, and human-baited double-net traps) targeting different development stages (i.e., egg, larvae, pupae, and adults) of *Aedes* mosquitoes during the dry and rainy seasons. Due to logistic limitations, our study only focused on *Aedes* mosquito dwelling up to 2 m above ground, and the anthropophagic populations that are active between 04:00 a.m. and 08:00 p.m. Some canopy-dweller [29], nighttime-biter [30, 31], and zoophilic [32] *Aedes* species were probably missed by the current sampling techniques. A vertical stratification study, circadian (24-hour period) sampling design, and animal-baited trapping could possibly provide deeper insight into the ecology of *Aedes* mosquitoes living in the canopies, darkness-dependent biting, and zoophagic behaviors, respectively.

Third, from a reductionist view, we found compositional differences in *Aedes* species among the landscape covers, suggesting ecologically filtering effects of land-use changes on *Aedes* mosquito communities, as observed in arthropods [33]. Bernues-Baneres et al. [34] have observed variations in faunistic diversity of mosquitoes according to the typology of land-covers in Spain. Because of their high sensitivity to environmental changes, mosquitoes have been suggested as bio-indicators of forest degradation level in Brazil [35]. In our study area, *Aedes* species were absent in oil palm monocultures, while they were abundantly present in polyculture environment and rural housing areas. This may suggest the displacement of *Aedes* mosquitoes vectors primarily from the forested areas transformed into oil palm plantations toward preserved rainforest, the polyculture, and rural housing areas for searching alternative breeding sites [36, 37], and blood-food sources [21]. In the first possible scenario, under the increased pressure exerted by *Aedes* mosquito populations, they become highly abundant during the rainy season on the hosts and breeding sites available in the preserved rainforest. The ecologic *Aedes*-rainforest balance is probably interrupted, and hence, leading to the diffusion of forest-dwelling anthropozoophilic *Aedes* species toward the rural human-inhabited areas. Similar findings have been reported in rural areas of Senegal, where *Aedes* vectors have invaded villages from surrounding landscapes and the risk of arboviral infection became highest at the edges of the villages [29]. These wild *Aedes* species that have both horizontal/oral and vertical/transovarial transmission competences for arbovirus probably transmit viruses that they have previously taken from forest-dwelling animals to villagers thus linking the jungle/sylvatic cycles to emergence/rural cycles [12, 20, 21]. Alternatively, the second scenario is that people working in polyculture could be bitten by a virus-infected *Aedes* mosquito, which might carry the virus to rural housing areas that are already colonized by potential competent vectors [20]. These competent vectors may disseminate viruses among the populations. Both scenarios are expected to increase yellow fever and dengue emergence and re-emergence risks, especially since they do not exclude mutually [20], because people live in close proximity to wildlife.

Fourth, *Aedes* mosquitoes still appear to show diverse and atypical breeding patterns across macro- and microhabitats leading to horizontal stratification among species with lack of *Aedes* mosquitoes in the oil palm monocultures and strong colonization of the other macrohabitats (i.e., rainforest, polyculture, and rural housing areas). These findings corroborate previous results showing that land-use changes affect the ecology of immature *Aedes* mosquitoes in the United States of America [2] and in rural areas of Senegal [7]. Ferraguti et al. [3] have reported that mosquito richness is higher in natural areas compared to anthropized areas. Polyculture areas have more positive effects on the abundance and species richness of terrestrial arthropod than monocultures in oil palm production landscapes in Peninsular Malaysia [5, 38]. Indeed, oil palm plantations alter ecosystem functioning [39], and reduce species richness and abundance compared with forested areas [40] due to the losses of habitats and hosts [5, 6]. Moreover, the drastic decline in *Aedes* species in oil palm monocultures could probably be exacerbated by multiple and intense uses of chemical products such as insecticides and herbicides for crop protection [19]. *Aedes* species have adapted alternatively their oviposition and blood-feeding behaviors to anthropogenic habitats and hosts that are available in the polyculture and rural housing areas [7]. Polyculture still had naturally-occurring microhabitats (i.e., tree and bamboo holes), developed multiple agriculturally-occurring microhabitats (i.e., crop fruit husks, flower, sheathing leaf axils, and cultivated plant holes), and received several man-made containers (i.e., crop collection containers, and discarded containers). Indeed, people discarded high numbers of containers such as old tires, parts of vehicles and machines in the maintenance of oil palm plantations, tarps, cans, and other worn items in surrounding polycultures since people live in close proximity to their smallholdings. Additionally, urbanized housing areas are incriminated to replace natural microhabitats (e.g., tree holes, bamboo) by artificial microhabitats (e.g., tires, discarded containers, and water storage containers), increase in the number of microhabitats expose breeding sites to a higher magnitude of solar radiation and enhance the population size of *Aedes* mosquitoes [41]. In such areas, containers used to provide water for poultry husbandry during the dry season were found to be highly infested with *Ae*. *aegypti* larvae, as observed in bird cages in Malaysia [9]. Anthropogenic environments also act as limiting factors for *Aedes* mosquito predators (e.g., *Eretmapodites* spp. and *Toxorhynchites* spp.) [4]. Hence, *Aedes* species that uniquely oviposit in natural containers (e.g., tree holes), may lay more fragile and desiccation-sensitive eggs. Rainwater is needed for hatching eggs, thus influencing oviposition behaviors [4, 7]. Of note, *Aedes* species need microbial inputs from predation as food sources for their offspring [2], and wild animal hosts as blood-meals for the adult females [32]. These features probably restricted certain *Aedes* species to the rainforest [4, 7]. Indeed, the specialists that are strictly ecologic demanding remain confined to particular ecotopes (e.g., rainforest), while the generalists (i.e., *Ae*. *aegypti*) might spread and colonize more diverse environments [4, 7]. However, *Ae*. *aegypti* mosquitoes seem to prefer anthropically altered areas rather than natural landscapes [4]. All these biotic and abiotic factors interact with rainfalls that habitually ensure the flooding of breeding sites to induce significant variations in the abundance and distribution of *Aedes* mosquito species, all of which may link the different possible arbovirus transmission cycles and increase exposure of human populations to arbovirus-risks [12].

Finally, *Aedes* mosquito females seem to exhibit similarities and dissimilarities in host-seeking behaviors between the types of land-cover that acted as a series of ecologic filters [33]. *Aedes* mosquitoes were seeking for humans in every land-cover type studied here, except for the oil palm monoculture. Moreover, the vectors displayed low preference for feeding on humans in the rainforest. Host-seeking activities were higher in both polyculture and rural housing areas, and biting activity showed one peak in the morning and one peak in the evening. However, biting cycles were interrupted between 10:00 a.m. and 02:00 p.m. in the rural housing areas and maintained in the polyculture. The unexpected ecologic variations in *Aedes* biting behavior suggest a complex pattern of arbovirus transmission in the large-scale development of oil palm-planted landscapes. Such outstanding spillovers might be attributable to the adaptation of *Aedes* species to land-use patterns, and human activities and movements. In fact, the absence of aggressive *Aedes* females in oil palm monoculture could be explained by the losses of their habitats and animal hosts [6], while the disinterest of rainforest-dwelling vectors into feeding on humans could be due to their preference to feed on wild animals [32]. When the vector aggressiveness peaked, in the early morning and in the evening, humans are generally within housing areas suggesting that high exposures to arboviruses occur in the villages [21, 28]. The interruption of host-seeking activities of *Aedes* females coincided with the migration of workers to the industrial oil palm farming and other people to their own smallholdings. Such an accordance of malaria vector behaviors to human movements has been reported in rubber plantations in Thailand [42]. The gap observed in host-seeking activities also corresponded to the sunlight intensity in the rural housing areas that are directly exposed to solar radiation due to the lack of natural vegetation coverage. As observed in poikilothermic animals, including insects [43], *Aedes* host-seeking behavior was probably most affected by the sun in the housing area. Conversely, the continuous biting cycles of *Aedes* females in polyculture could be explained by the permanent presence of workers that may habitually serve as blood-food sources [42], and the shade provided by the abundance of vegetation coverage that probably reduces the negative effects of sunlight radiation on host-searching activities. The surprising darkness-biting activities could be interpreted as residual biting activities of *Aedes* mosquitoes that feed at night on wild animals [21, 29, 32]. The nocturne biting activities of the well-known daytime *Aedes* mosquitoes has been reported on *Ae*. *aegypti* in Côte d’Ivoire [30] and *Ae*. *albopictus* in Cameroon [31]. The extent of such atypical host-seeking activity rhythm observed in our study region could have important epidemiologic implications, and needs to be analyzed in greater depth, over longer times and larger scales.

We conclude that in the southeastern part of Côte d’Ivoire, agricultural land-use has changed as a result of transforming rainforest into oil palm monocultures, which significantly influences the composition, distribution, oviposition patterns, and host-seeking behavior of *Aedes* mosquito species. In turn, there is a lack of *Aedes* mosquitoes in oil palm monocultures and a strong colonization of polyculture and rural housing areas. Hence, humans are increasingly exposed to *Aedes* bites and arbovirus risk around their homes and farming plots. The polyculture and the rural housing ecotopes thus represent priority areas for vector control and surveillance. In oil palm-planted areas, arboviral disease control strategy should encompass integrated approaches, including landscape ecology and epidemiology, and ecotope-based vector control.

## Supporting information

S1 Fig Different macro- and microhabitat types sampled for *Aedes* mosquitoes in oil palm-dominated landscapes in southeastern Côte d’Ivoire. Potential habitats of *Aedes* mosquitoes are stratified into two habitat types: macrohabitats (A-D), and microhabitats (E-P). The habitat type often reflects the name of the habitats and the categories include habitats that provide comparable *Aedes* mosquito habitats. The macrohabitats are divided into four ecological blocks: A: Rainforest that was preserved dense forest hosting several plant species of trees, creepers, and bamboo, and animals; B: Polyculture that covered a mixture of cultivated plants such as oil palm tree, rubber, taro, banana, coconuts, and native trees; C: Oil palm monoculture that was covered uniquely with industrial oil palm trees; and D: rural-housing areas that are characterized by human-inhabited space. The microhabitats (E-P) were summarized into: Naturally-occurring microhabitats (E-H) that comprised E: Natural tree hole, F: Bamboo hole, G: Natural plant leaf, and H: Other natural microhabitats; Agriculturally-occurring microhabitats (I-L) that were composed of: I: Crop fruit husk, J: Crop flower, K: Crop leaf, and L: Cultivated plant hole; and Man-made microhabitats (M-P) that represented: M: Crop collection container, N: Husbandry watering container, O: Discarded container, and P: Household water container. Containers were categorized as “other natural microhabitats”, such as snail shells and rock holes.
(TIF)

S2 Fig Standardized methods used for sampling different life stages of *Aedes* mosquitoes in the study area. A: Bamboo-ovitrap, B: Metallic-ovitrap, C: Larval survey, D: Human-baited double net trap.
(TIF)

S3 Fig *Aedes* mosquito species occurrence among the microhabitats in different macrohabitats in southeastern Côte d’Ivoire surveyed from January to December 2014. Error bars represent the standard error (SE). NOM: naturally-occurring microhabitat, AOM: agriculturally-occurring microhabitat, MMM: man-made microhabitat.
(TIF)

S4 Fig Relative proportions (%) of the different types of microhabitats among *Aedes*-positive microhabitats among the macrohabitats in southeastern Côte d’Ivoire surveyed from January to December 2014. Error bars represent the standard error (SE). NOM: naturally-occurring microhabitat, AOM: agriculturally-occurring microhabitat, MMM: man-made microhabitat.
(TIF)

S5 Fig Monthly variations in *Aedes* mosquito species occurrence among the microhabitats in different macrohabitats in southeastern Côte d’Ivoire surveyed from January to December 2014. Error bars represent the standard error (SE).
(TIF)

S6 Fig Monthly variations in different types of microhabitats among *Aedes*-positive microhabitats among the macrohabitats in southeastern Côte d’Ivoire surveyed from January to December 2014. Error bars represent the standard error (SE).
(TIF)

S1 Table Outputs of data analysis on positivity rates of *Aedes* collected as eggs using bamboo-ovitraps in oil palm-dominated landscapes in southeastern Côte d’Ivoire surveyed from January to December 2014. Results are the outputs of the generalized linear mixed model (GLMM) procedures. Results are considered significant for p-values <0.05.
(DOCX)

S2 Table Outputs of data analysis on mean numbers of *Aedes* collected as eggs using bamboo-ovitraps in oil palm-dominated landscapes in southeastern Côte d’Ivoire surveyed from January to December 2014. Results are the outputs of the generalized linear mixed model (GLMM) procedures. Results are considered significant for p-values <0.05.
(DOCX)

S3 Table Outputs of data analysis comparing the mean numbers of *Aedes* eggs using metallic-ovitraps in rainforest to the other macrohabitats in oil palm-dominated landscapes in southeastern Côte d’Ivoire sampled from January to December 2014. Results are the outputs of the generalized linear mixed model (GLMM) procedures. Result are considered significant for p-values <0.05.
(DOCX)

S4 Table Outputs of data analysis comparing the mean numbers of *Aedes* eggs collected using metallic-ovitraps in polyculture with the other macrohabitats in oil palm-dominated landscapes in southeastern Côte d’Ivoire samples from January to December 2014. Results are the outputs of the generalized linear mixed model (GLMM) procedures. Results are considered significant for p-values <0.05.
(DOCX)

S5 Table Outputs of data analysis on the mean numbers *Aedes* females’ host-seeking activities in oil palm-dominated landscapes in southeastern Côte d’Ivoire sampled from January to December 2014. Results are the outputs of the generalized linear mixed model (GLMM) procedures. Results are considered significant for p-values <0.05.
(DOCX)

S6 Table Synthesis of how land-use changes might affect the dynamics of *Aedes* mosquitoes in oil palm-dominated areas in southeastern Côte d’Ivoire. **—**: very low risk,—: low risk, +: high risk, ++: very high risk; %: percentage; SE: standard error of the mean. Host-seeking activity is expressed as the mean numbers of *Aedes* females collected per human-baited double-net trap. The unit of host-seeing activity is female/person/day. Overall, there was a lack of *Aedes* microhabitats and species in the oil palm monoculture resulting in very low arbovirus risk. In contrast, the highest abundance of *Aedes* mosquitoes was found in the polyculture where arbovirus risk is expected to be very high. The highest species richness was observed in the rainforest where the preference of *Aedes* females to feed on humans was low. The rural housing areas and the whole study area hosted substantial numbers of *Aedes* mosquitoes and arbovirus risk is expected to be high in rural housing area and moderate in the whole study area.
(DOCX)
