# Supplementary figures and images for: Effect of land-use changes on the abundance, distribution, and host-seeking behavior of Aedes arbovirus vectors in oil palm-dominated landscapes, southeastern Côte d’Ivoire
Source: PLoS One. 2017 Dec 7;12(12):e0189082. doi: 10.1371/journal.pone.0189082 (PMC5720743; doi:10.1371/journal.pone.0189082)

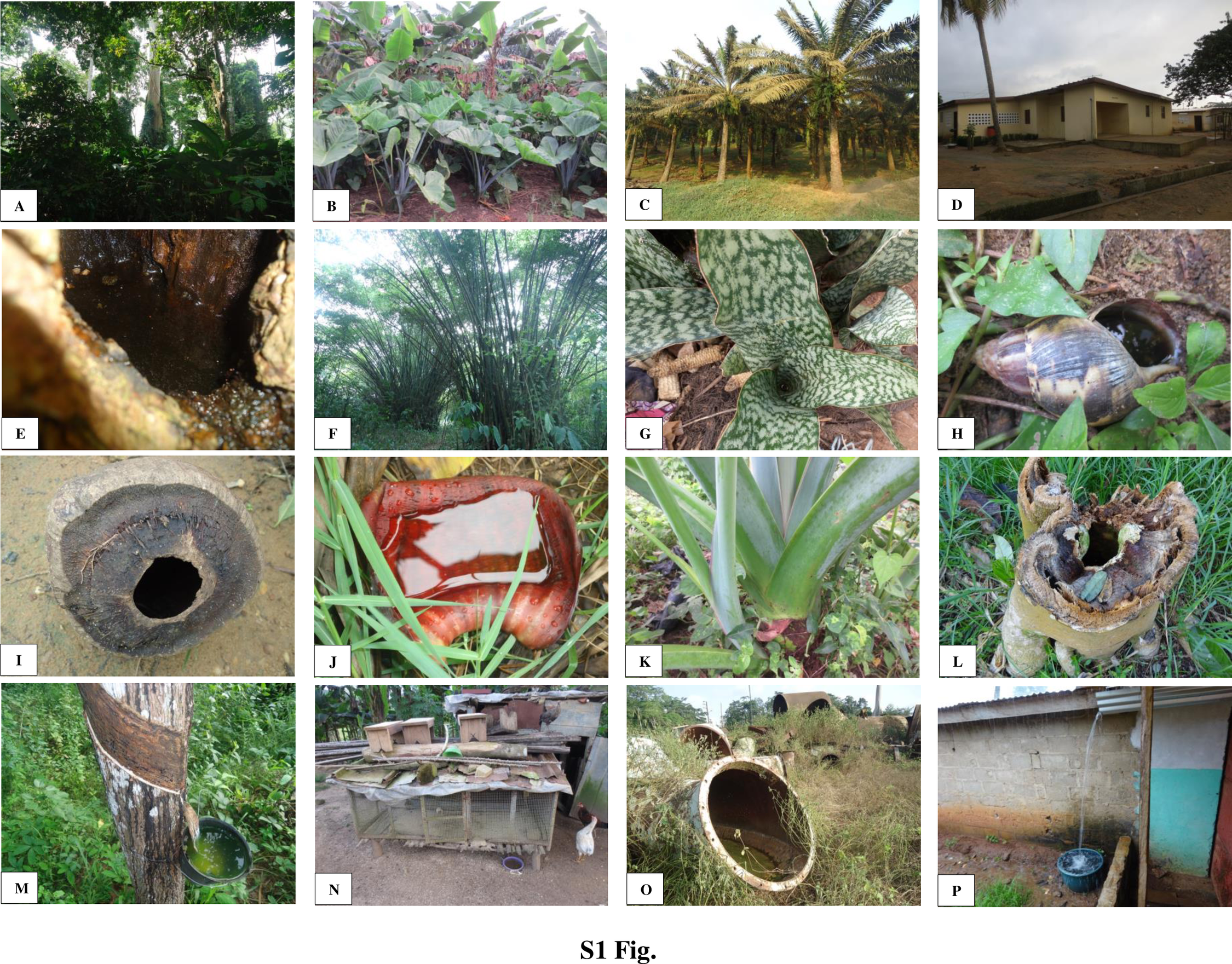

Supplement: S1 Fig — Potential habitats of Aedes mosquitoes are stratified into two habitat types: macrohabitats (A-D), and microhabitats (E-P). The habitat type often reflects the name of the habitats and the categories include habitats that provide comparable Aedes mosquito habitats. The macrohabitats are divided into four ecological blocks: A: Rainforest that was preserved dense forest hosting several plant species of trees, creepers, and bamboo, and animals; B: Polyculture that covered a mixture of cultivated plants such as oil palm tree, rubber, taro, banana, coconuts, and native trees; C: Oil palm monoculture that was covered uniquely with industrial oil palm trees; and D: rural-housing areas that are characterized by human-inhabited space. The microhabitats (E-P) were summarized into: Naturally-occurring microhabitats (E-H) that comprised E: Natural tree hole, F: Bamboo hole, G: Natural plant leaf, and H: Other natural microhabitats; Agriculturally-occurring microhabitats (I-L) that were composed of: I: Crop fruit husk, J: Crop flower, K: Crop leaf, and L: Cultivated plant hole; and Man-made microhabitats (M-P) that represented: M: Crop collection container, N: Husbandry watering container, O: Discarded container, and P: Household water container. Containers were categorized as “other natural microhabitats”, such as snail shells and rock holes. (TIF) [file pone.0189082.s001.tif]

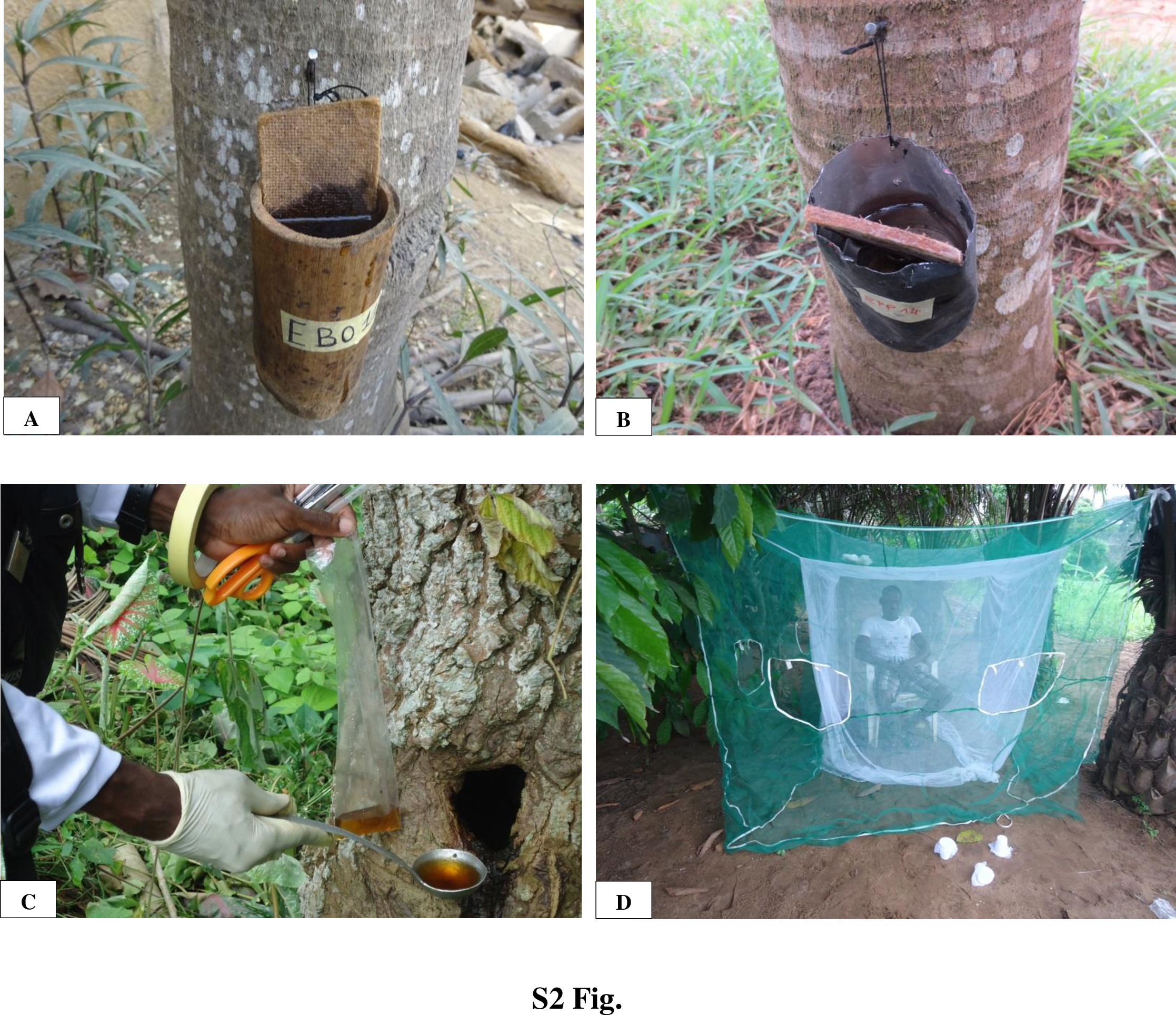

Supplement: S2 Fig — A: Bamboo-ovitrap, B: Metallic-ovitrap, C: Larval survey, D: Human-baited double net trap. (TIF) [file pone.0189082.s002.tif]

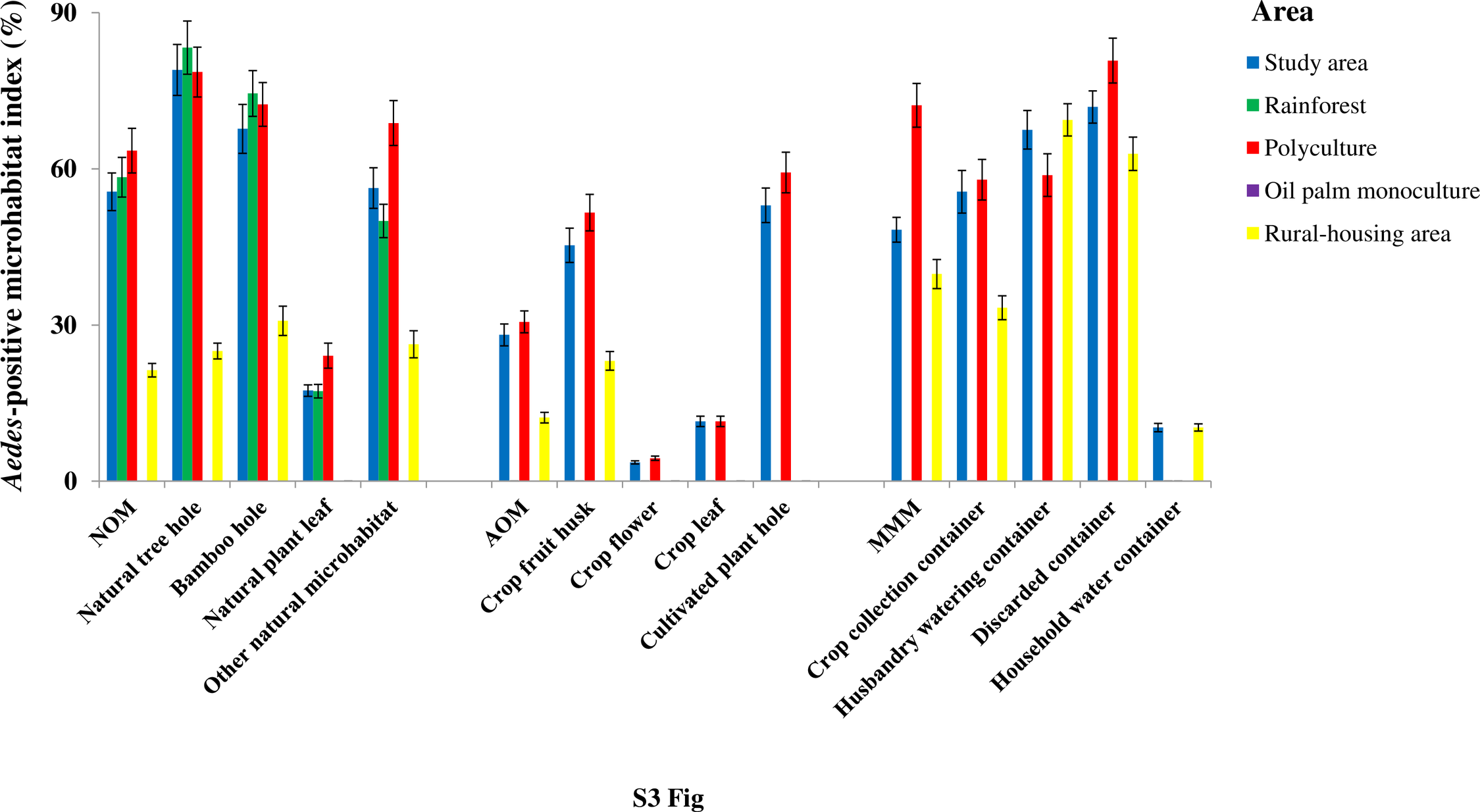

Supplement: S3 Fig — Error bars represent the standard error (SE). NOM: naturally-occurring microhabitat, AOM: agriculturally-occurring microhabitat, MMM: man-made microhabitat. (TIF) [file pone.0189082.s003.tif]

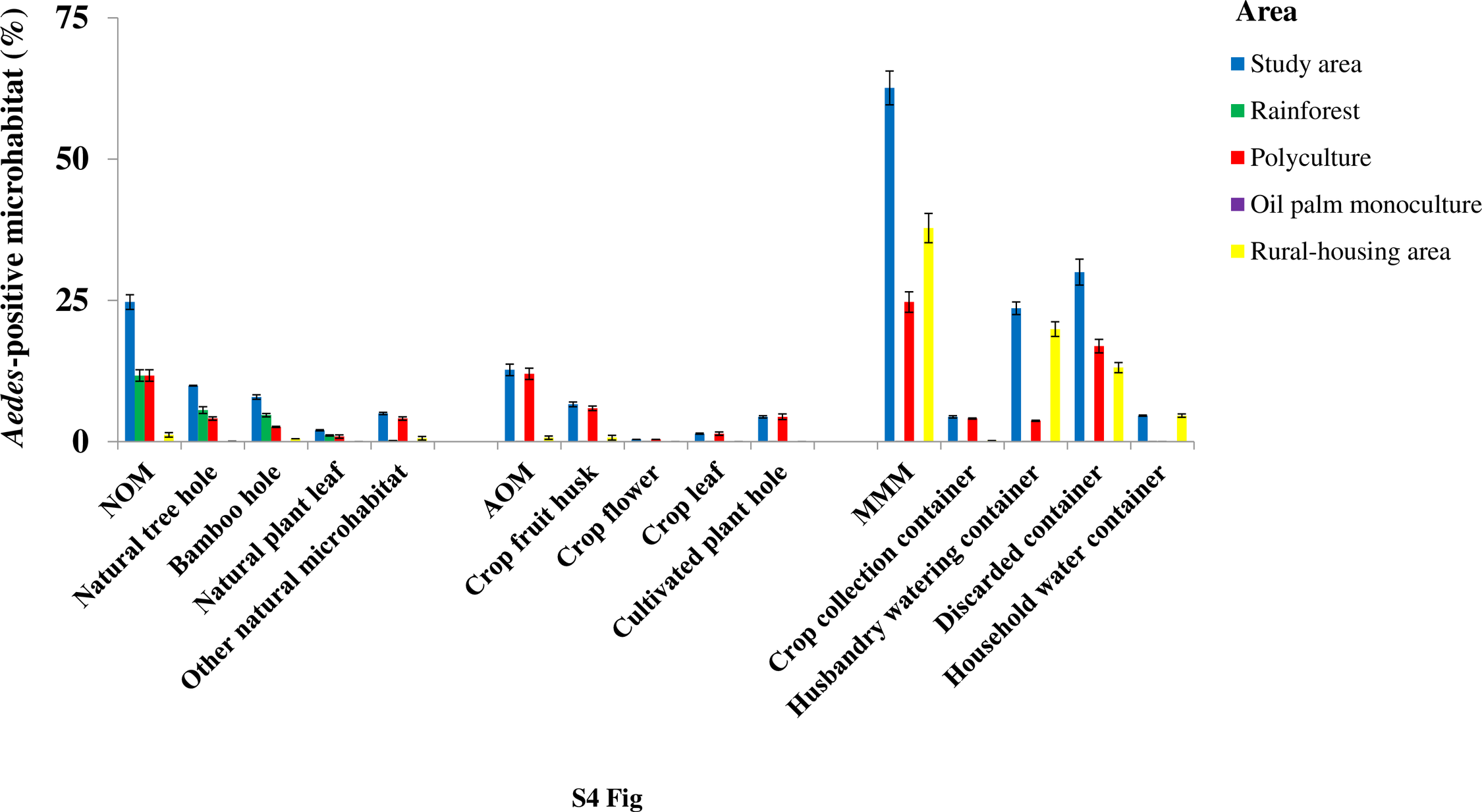

Supplement: S4 Fig — Error bars represent the standard error (SE). NOM: naturally-occurring microhabitat, AOM: agriculturally-occurring microhabitat, MMM: man-made microhabitat. (TIF) [file pone.0189082.s004.tif]

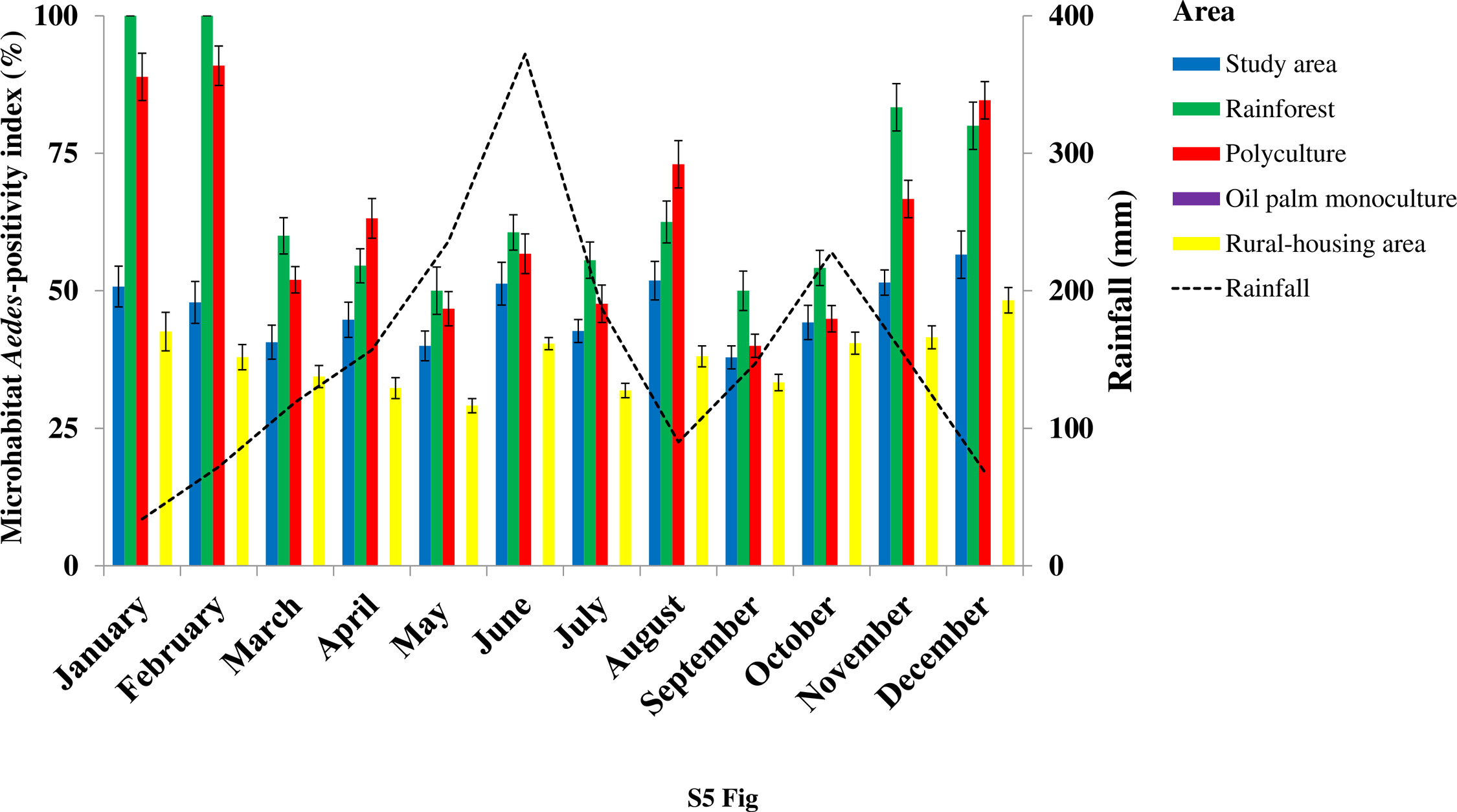

Supplement: S5 Fig — Error bars represent the standard error (SE). (TIF) [file pone.0189082.s005.tif]

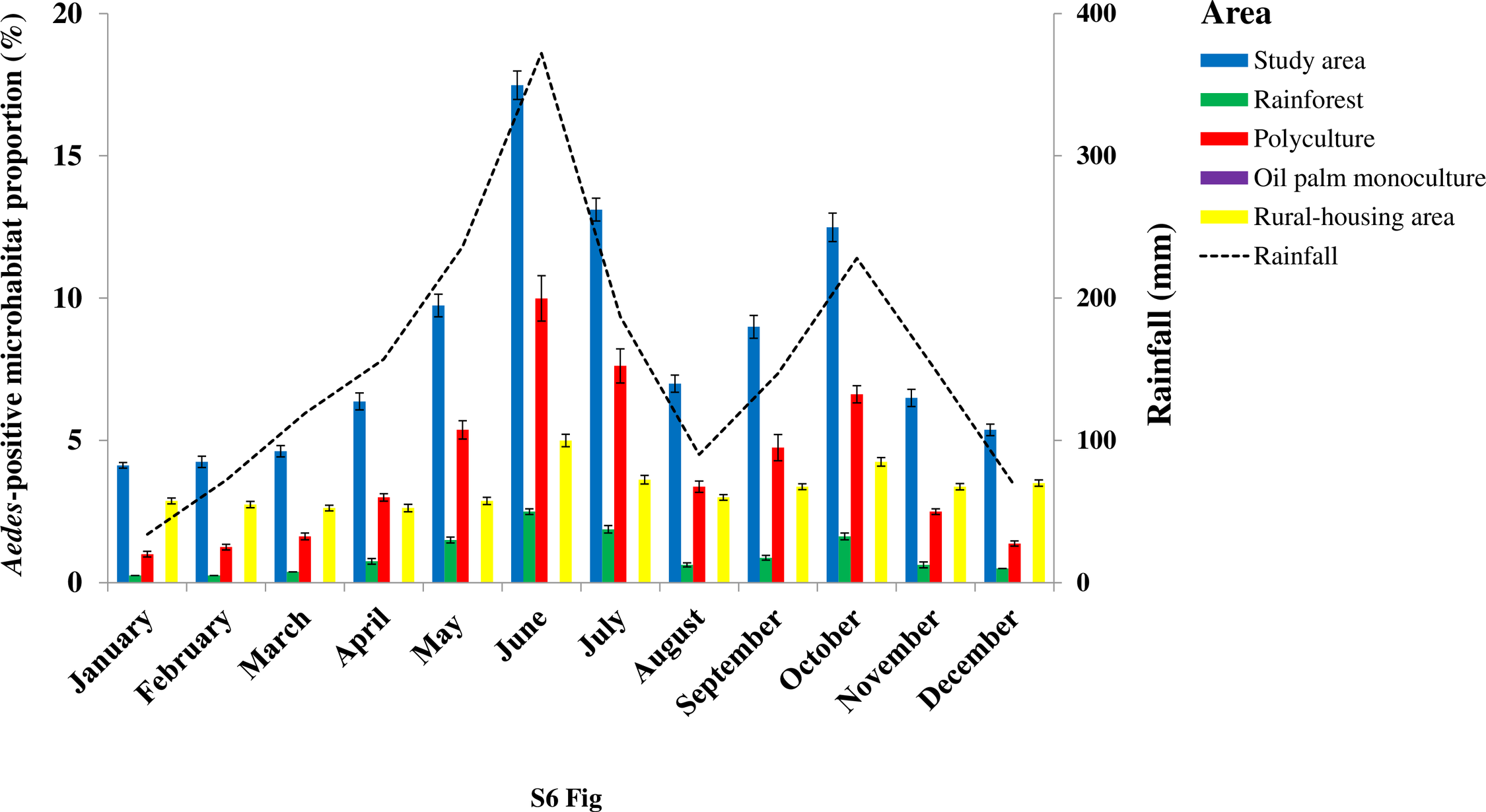

Supplement: S6 Fig — Error bars represent the standard error (SE). (TIF) [file pone.0189082.s006.tif]
