# Supplementary material for: Effect of land-use changes on the abundance, distribution, and host-seeking behavior of Aedes arbovirus vectors in oil palm-dominated landscapes, southeastern Côte d’Ivoire
Source: PLoS One. 2017 Dec 7;12(12):e0189082. doi: 10.1371/journal.pone.0189082 (PMC5720743; doi:10.1371/journal.pone.0189082)
Supplement: S1 Table — Results are the outputs of the generalized linear mixed model (GLMM) procedures. Results are considered significant for p-values <0.05. (DOCX) [file pone.0189082.s007.docx]

**S1 Table.** Outputs of data analysis on positivity rates of *Aedes* collected as eggs using bamboo-ovitraps in oil palm-dominated landscapes in southeastern Côte d’Ivoire from January to December 2014
