# Supplementary material for: Effect of land-use changes on the abundance, distribution, and host-seeking behavior of Aedes arbovirus vectors in oil palm-dominated landscapes, southeastern Côte d’Ivoire
Source: PLoS One. 2017 Dec 7;12(12):e0189082. doi: 10.1371/journal.pone.0189082 (PMC5720743; doi:10.1371/journal.pone.0189082)
Supplement: S5 Table — Results are the outputs of the generalized linear mixed model (GLMM) procedures. Results are considered significant for p-values <0.05. (DOCX) [file pone.0189082.s011.docx]

**S5 Table.** Outputs of data analysis on the mean numbers *Aedes* females’ host-seeking activities in oil palm-dominated landscapes in southern Côte d’Ivoire from January to December 2014
