# Supplementary material for: Effect of land-use changes on the abundance, distribution, and host-seeking behavior of Aedes arbovirus vectors in oil palm-dominated landscapes, southeastern Côte d’Ivoire
Source: PLoS One. 2017 Dec 7;12(12):e0189082. doi: 10.1371/journal.pone.0189082 (PMC5720743; doi:10.1371/journal.pone.0189082)
Supplement: S6 Table — —: very low risk,—: low risk, +: high risk, ++: very high risk; %: percentage; SE: standard error of the mean. Host-seeking activity is expressed as the mean numbers of Aedes females collected per human-baited double-net trap. The unit of host-seeing activity is female/person/day. Overall, there was a lack of Aedes microhabitats and species in the oil palm monoculture resulting in very low arbovirus risk. In contrast, the highest abundance of Aedes mosquitoes was found in the polyculture where arbovirus risk is expected to be very high. The highest species richness was observed in the rainforest where the preference of Aedes females to feed on humans was low. The rural housing areas and the whole study area hosted substantial numbers of Aedes mosquitoes and arbovirus risk is expected to be high in rural housing area and moderate in the whole study area. (DOCX) [file pone.0189082.s012.docx]

| **Macrohabitat (Land-cover)** | **Rainforest** | **Polyculture** | **Oil palm monoculture** | **Rural housing areas** | **Study area** |
| --- | --- | --- | --- | --- | --- |
| Bamboo-ovitrap | 43.6 | 50.6 | 0.0 | 45.4 | 35.0 |
| Metallic-ovitrap | 44.2 | 67.4 | 0.6 | 56.2 | 41.9 |
| Microhabitat positivity (%) | 58.4 | 52.6 | 0 | 37.2 | 45.6 |
| Naturally-occurring (%) | 100 | 51.0 | 0 | 95.0 | 55.6 |
| Agriculturally-occurring (%) | 0 | 24.8 | 0 | 1.9 | 28.1 |
| Made-made (%) | 0 | 24.2 | 0 | 3.1 | 48.3 |
| Species richness (no of species) | 11 | 10 | 1 | 8 | 11 |
| Abundance (%) | 11.0 | 60.9 | 0.01 | 28.0 | 100 |
| Host-seeking activity (f/p/d) | 0.62 ± 0.06 | 21.48 ± 0.12 | 0 | 4.48 ± 0.10 | 2.76 ± 0.07 |
| Ratio (macrohabitat/rainforest) | 1 | 34.6 | 0 | 7.2 | 4.5 |
| Arbovirus-risk | - | ++ | -- | + | + |

**S6 Table. Synthesis of how land-use changes affect the dynamics of *Aedes* mosquitoes in oil palm-dominated areas in southeastern Côte d’Ivoire**

f/p/d: female/person/day. -- : very low risk, - : low risk, + : high risk, ++ : very high risk
